# Longitudinal Analysis of the Microbiome and Metabolome in the 5xfAD Mouse Model of Alzheimer’s Disease

**DOI:** 10.1128/mbio.01794-22

**Published:** 2022-12-05

**Authors:** Sage J. B. Dunham, Katelyn A. McNair, Eric D. Adams, Julio Avelar-Barragan, Stefania Forner, Mark Mapstone, Katrine L. Whiteson

**Affiliations:** a Department of Molecular Biology and Biochemistry, University of California Irvine, Irvine, California, USA; b Department of Computational Science, University of California Irvine, Irvine, California, USA; c Institute for Memory Impairments and Neurological Disorders (UCI MIND), University of California Irvine, Irvine, California, USA; d Department of Neurology, University of California Irvine, Irvine, California, USA; University of Michigan-Ann Arbor

**Keywords:** Alzheimer’s disease, AD, MODEL-AD, microbiome, metabolome, gut-brain axis, *Turicibacter*, serotonin

## Abstract

Recent reports implicate gut microbiome dysbiosis in the onset and progression of Alzheimer’s disease (AD), yet studies involving model animals overwhelmingly omit the microbial perspective. Here, we evaluate longitudinal microbiomes and metabolomes from a popular transgenic mouse model for familial AD (5xfAD). Cecal and fecal samples from 5xfAD and wild-type B6J (WT) mice from 4 to 18 months of age were subjected to shotgun Illumina sequencing. Metabolomics was performed on plasma and feces from a subset of the same animals. Significant genotype, sex, age, and cage-specific differences were observed in the microbiome, with the variance explained by genotype at 4 and 18 months of age rising from 0.9 to 9% and 0.3 to 8% for the cecal and fecal samples, respectively. Bacteria at significantly higher abundances in AD mice include multiple *Alistipes* spp., two *Ligilactobacillus* spp., and *Lactobacillus* sp. P38, while multiple species of *Turicibacter*, Lactobacillus johnsonii, and Romboutsia ilealis were less abundant. *Turicibacter* is similarly depleted in people with AD, and members of this genus both consume and induce the production of gut-derived serotonin. Contradicting previous findings in humans, serotonin is significantly more concentrated in the blood of older 5xfAD animals compared to their WT littermates. 5xfAD animals exhibited significantly lower plasma concentrations of carnosine and the lysophospholipid lysoPC a C18:1. Correlations between the microbiome and metabolome were also explored. Taken together, these findings strengthen the link between *Turicibacter* abundance and AD, provide a basis for further microbiome studies of murine models for AD, and suggest that greater control over animal model microbiomes is needed in AD research.

## INTRODUCTION

Alzheimer’s disease (AD) remains a debilitating problem for the health and wellbeing of people all over the world. At the time of this writing, an estimated 6.2 million Americans aged 65 or older are living with clinical (symptomatic) AD, and millions more have preclinical AD (1, 2). Approximately 1 in every 5 American women and 1 in 10 American men can expect to develop AD-related dementia in their lifetimes (1, 2). This grim health outlook is accompanied by the staggering cost borne by caregivers and taxpayers. In 2021 alone, the health care expenditure on AD in the US was estimated to be more than $350 billion, with more than $230 billion in direct costs billed to Medicare and Medicaid. An additional estimated $250 billion in unpaid care is provided by friends and family (1, 2). This devastating human and financial toll demands an ambitious research effort.

Researchers are working to untangle the mystery of AD from almost every conceivable angle. We now understand much about the physiological hallmarks of the disease, including the characteristic brain changes (3) and many of the risk factors for AD, including genetic- (4) and environmental- or lifestyle-based (5). However, despite this extensive research, a cure remains tragically elusive. Of the six drugs currently approved for the treatment of AD, five treat the symptoms of the disease but do not slow its progression. The sole exception is the controversial monoclonal antibody-based drug “aducanumab” that is reported to reduce the accumulation of amyloid beta (Aβ) plaques (6–9).

One reason a cure has remained elusive is an insufficient recapitulation of the neuropathology and behavioral aspects of AD in animal models. Scientists have developed more than 100 genetically engineered mouse lines that express specific aspects of AD clinicopathology, but, as valuable as they are, none of these models adequately recapitulate many important aspects of the disease (10, 11). This is especially the case for late onset AD, which accounts for most cases (12). In response to this challenge, researchers have formed a National Institute on Aging (NIA) funded consortium, the Model Organism Development & Evaluation for Late-Onset Alzheimer’s Disease (MODEL-AD), to develop better animal models for AD (13).

Awareness is growing around the role that the host microbial community plays in AD pathology (14–18), including the interrelationship between inflammation, microbial exposure, and AD development (19–21). A slate of studies have shown that antibiotics improve behavioral symptoms and reduce amyloid plaque pathology (16, 22, 23), and germfree murine models for AD exhibit reduced neuropathology relative to those with a normal microbiome (24). The human gut microbiome composition has been associated with AD in several studies (25, 26), and blood bile acid signatures (a readout of both host and microbiome metabolism) have been correlated with cognitive decline in AD (27). Despite this increased evidence, those developing and validating animal models often neglect consideration of the microbiome.

**FIG 1:**
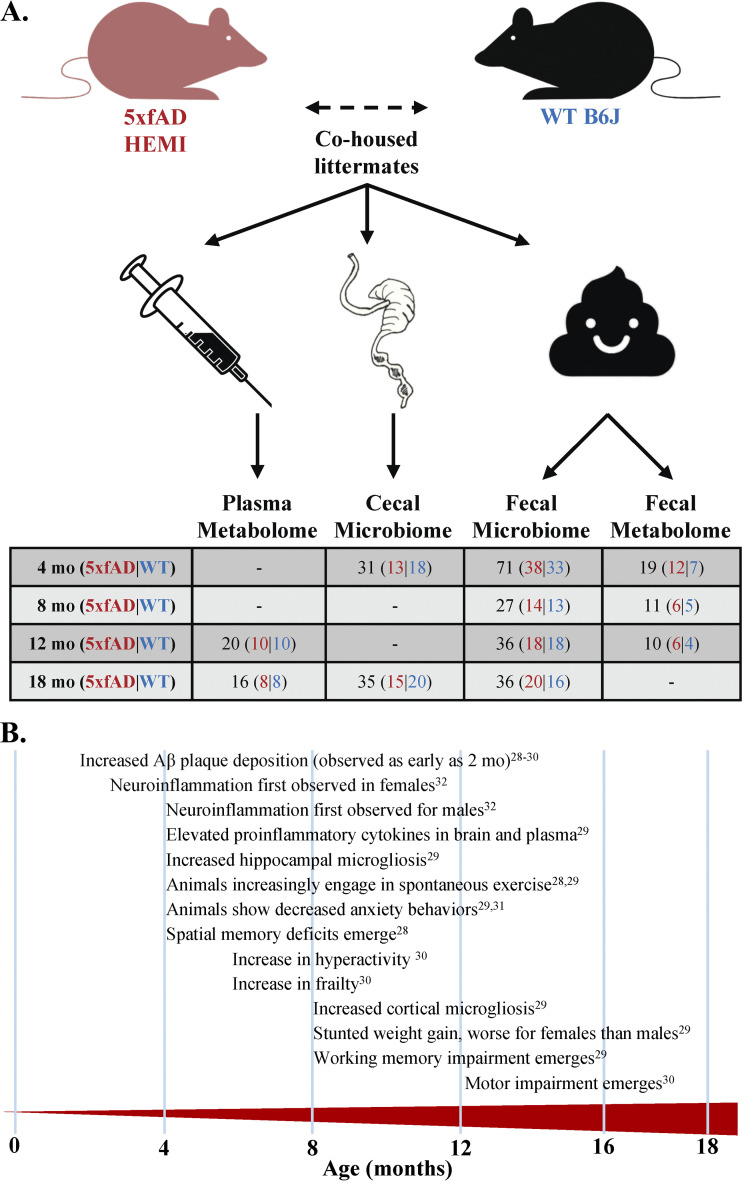
Experimental design and overview of AD pathology in aging 5xfAD mice. (A) Fecal samples were collected at 4, 8, 12, and 18 months of age for shotgun metagenomics and metabolomics (4, 8, and 12 months only). Cecal samples were collected at 4 and 18 months for shotgun metagenomics. Blood plasma was harvested at 12 and 18 months of age for metabolomics. (B) 5xfAD mice exhibit many hallmarks of AD neuro- and behavioral-pathology, most of which are exacerbated by age. This includes the progressive deposition of Aβ plaques (28–30), motor impairment (30), neuroinflammation (29, 32), memory deficits (28, 29), and stunted weight gain (29).

Considering the mounting evidence, it is imperative that we examine model animals from the perspective of the gut microbiome. Here, we present a longitudinal characterization of the fecal and cecal microbiomes and metabolomes of a familial AD transgenic mouse model (5xfAD) in comparison to wild-type (WT) B6J mice See Fig. 1A for an overview of sampling. Originally introduced in 2006 (28), the 5xfAD mouse has become the gold-standard for familial AD research and is the basis for several emerging mouse lines. 5xfAD mice exhibit many features of AD, such as intraneuronal-amyloid aggregates, neurodegeneration, neuron loss, and impaired memory, with progressive changes emerging as early as 6 weeks of age (Fig. 1B) (28–32). Two recent MODEL-AD studies have comprehensively evaluated many important characteristics of the 5xfAD mouse, including behavior, cognition, and neuropathology (29, 30). Aside from a brief assessment by Oblak et al., the microbiome was left largely undiscussed (30). Here, we applied shotgun metagenomic sequencing and metabolomics to explore the microbes and metabolites that differ between WT and 5xfAD mice from 4 to 18 months of age. The associations between the microbiome and metabolome and the age, sex, and housing assignment of the mice were examined. We also examined the correlations between microbes and metabolites in both blood plasma and feces.

## RESULTS

### Shotgun metagenomic sequencing sample demographics.

Shotgun metagenomics were performed on 66 cecal samples from 4- and 18-month-old mice and on 170 fecal samples from 4-, 8-, 12-, and 18-month-old mice. The samples had a slightly higher representation of WT animals than 5xfAD animals (128 WT versus 108 5xfAD) and slightly more females than males (124 females versus 114 males). The average number of reads per sample for each cohort ranged from 1.1 to 1.9 million, with 18 to 28% of those reads aligning to a genome in the GenBank NT database.

### Overview of most abundant microbes.

**FIG 2:**
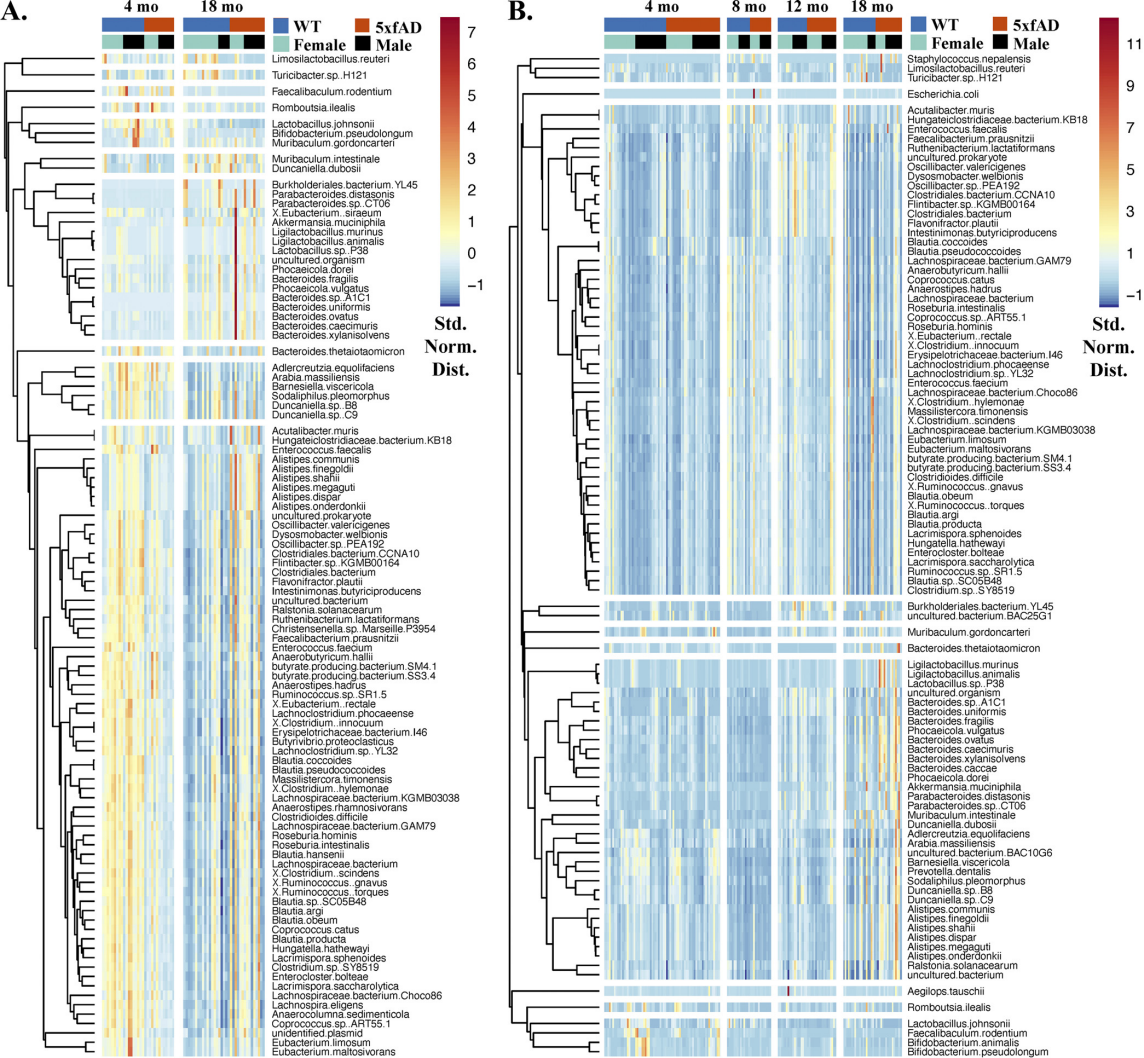
Clustered heatmaps of the 100 most abundant microbial species in the cecal (A) and fecal (B) samples, with each sample represented by a column and each species represented by a row. The color scale indicates abundance (standard normal distribution), with hierarchical clustering being used to group microbes with similar abundance trends. The hierarchical clustering of species is represented by the dendrograms to the left of each figure.

Clustered heat maps of the 100 most abundant microbial species in cecal (Fig. 2A) and fecal (Fig. 2B) samples showed many abundance variations with respect to both age and genotype. For example, the first three bacteria in the heat map for the cecal samples (Limosilactobacillus reuteri, *Turicibacter* sp. H121, and Faecalibaculum rodentium) had lower abundances in the 18-month 5xfAD samples, relative to both the 18-month WT samples and the 4-month samples of both genotypes. Those microbes in the seventh cecal cluster (comprised of 17 species from the genera *Burkholderiales*, *Akkermansia*, *Ligilactobacillus*, and *Bacteroides*, among others) were generally more abundant at 18 months of age than at 4 months of age. In the fecal samples (Fig. 2B), *T.* sp. H121 and L. reuteri were found together in the first cluster with Staphylococcus nepalensis, while *F. rodentium* was found in the third cluster. Organisms in the seventh cluster of the fecal samples had increased abundances in 18 month old mice. Among others, this cluster included multiple species from the genera *Ligilactobacillus*, *Bacteroides*, *Alistipes*, and *Duncaniella*. Microbial abundance differences with respect to sex were not readily apparent in this representation of the data.

### Alpha and beta diversity of the cecal and fecal microbiomes.

**FIG 3:**
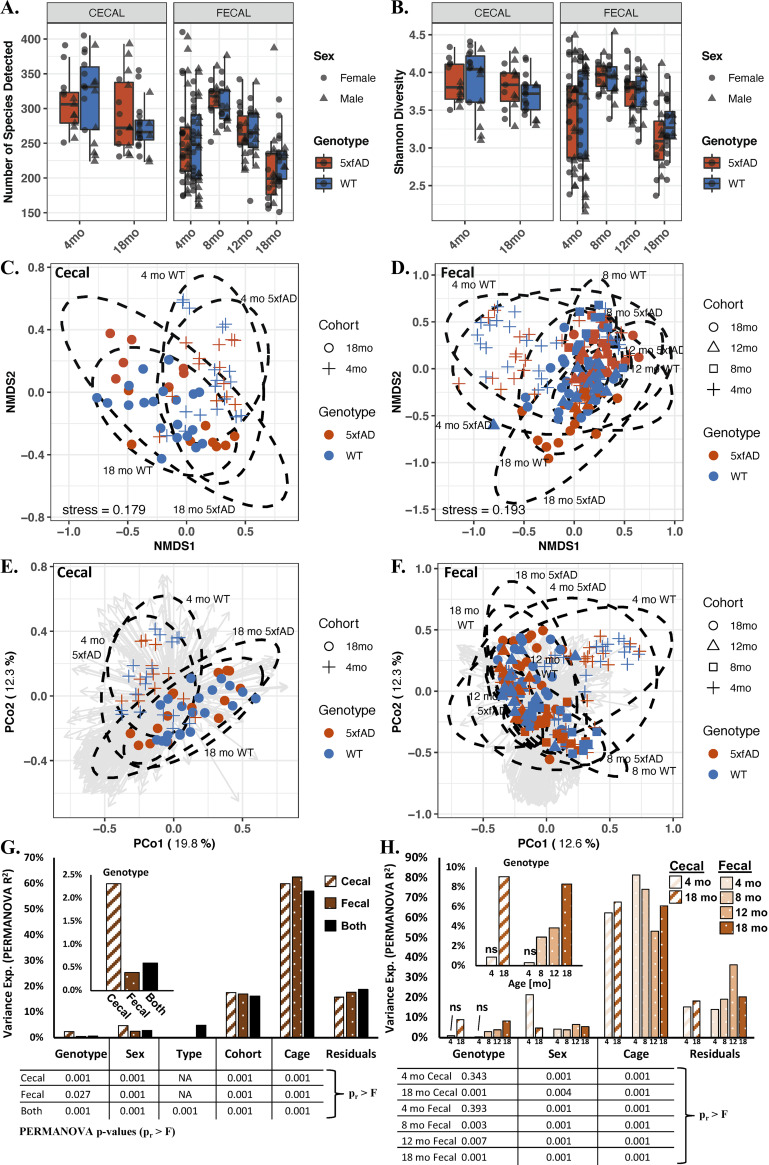
Alpha and beta diversities of the cecal and fecal microbiomes of 5xfAD and WT mice from 4 to 18 months of age. Shown are the species richness (A), Shannon diversity (B), nonmetric multidimensional scaling (NMDS) for the cecal (C) and fecal samples (D), PCoA the cecal (E) and fecal (F) samples, and variance explained by permutational multivariate analysis of variance (PERMANOVA) for all of the cohorts combined (G) and for each cohort considered separately (H). Alpha diversity (A and B) was not significantly different between genotypes, but it was significantly different with respect to age (richness: *P* < 0.05 for age, *P* > 0.05 for genotype via a two-way ANOVA; Shannon: *P* < 0.05 for age, *P* > 0.05 for genotype via a Kruskal-Wallis one-way ANOVA). The beta diversity, as visualized by NMDS (C and D) and a principal coordinates analysis (PCoA) (E and F) of the Bray-Curtis dissimilarity matrices, largely showed separation by age but not by genotype. PERMANOVA (G and H) shows that most of the variance in microbiome composition is attributed to housing assignment. All variables examined are significant, except for genotype in the 4-month samples (PERMANOVA *P* < 0.05), and the variance attributable to genotype increases with the age of the mice. The box plots in panels A and B show the median value +/− the first and third quartiles, with the whiskers showing the range or 1.5× the interquartile range, whichever is less. The analysis in Panels C–H was performed on the relative abundance data, with ellipses in C-F representing the 95% confidence interval for each age-genotype grouping. The arrows in panels E and F are PCoA loading eigenvectors, with each vector representing the direction and magnitude of a given microbe to the PCoA separation. The tabulated *P* values below the bar graphs in panels G and H were computed via PERMANOVA. More PERMANOVA details are provided in Table S1.

The richness (Fig. 3A) and the Shannon diversity (Fig. 3B) of the cecal microbiomes did not significantly differ with respect to age or genotype (*P* > 0.05 via a Kruskal-Wallis one-way ANOVA). For the fecal samples, both the richness and the Shannon diversity showed significant overall differences with respect to age but not with respect to genotype (via a two-way ANOVA). Both of the fecal alpha diversity metrics increased between 4 and 8 months of age and then successively decreased thereafter. Separating the cecal and fecal data based on sex (i.e., evaluating males and females separately) (Fig. S1A) also failed to uncover significant Shannon diversity differences with respect to genotype.

10.1128/mbio.01794-22.2 FIG S1 Alpha and beta diversity with males and females considered separately. (A) Shannon diversity of cecal samples. (B) Shannon diversity of fecal samples. (C) PCoA of female cecal samples only. (D) PCoA of female cecal samples only. (E) PCoA of male cecal samples only. (F) PCoA of male cecal samples only. No 5xfAD versus WT comparisons for Shannon diversity were significantly different (via *t* tests with *P* > 0.05 in all cases). Download FIG S1, PDF file, 0.3 MB. Copyright © 2022 Dunham et al. 2022 Dunham et al. https://creativecommons.org/licenses/by/4.0/ This content is distributed under the terms of the Creative Commons Attribution 4.0 International license.

10.1128/mbio.01794-22.1 TABLE S1 Permutational multivariate analysis of variance (PERMANOVA) of the model animal microbiomes corresponding to the main text Fig. 3G and H. Computations were performed in R using the “adonis” function in the Vegan package. Many variables were confounded, as littermates of the same sex often (but not always) shared the same cage. Download Table S1, PDF file, 0.03 MB. Copyright © 2022 Dunham et al. 2022 Dunham et al. https://creativecommons.org/licenses/by/4.0/ This content is distributed under the terms of the Creative Commons Attribution 4.0 International license.

Beta diversity, as evaluated through both nonmetric multidimensional scaling (NMDS) (Fig. 3C and D) and a principal coordinates analysis (PCoA) (Fig. 3E and F) of the Bray-Curtis dissimilarity matrix, showed general separation by age but not by genotype. The NMDS results exhibited high stress values (cecal = 0.179; fecal = 0.193) and a substantial overlap of the 95% confidence intervals for genotype, indicating weak associations. Inspection of the PCoA loadings (gray vectors in Fig. 3E and F) revealed separation arising from contributions of many species (as opposed to a few species driving differences between groups). The PCoA of the males and females separately (Fig. S1B–E) showed more distinct separation by age but not by genotype.

Permutational multivariate analysis of variance (PERMANOVA) was performed in multiple ways to explore significant differences in beta diversity, including for: cecal samples only (dashed bars in Fig. 3G), fecal samples only (spotted bars in Fig. 3G), cecal and fecal samples together (“both” in Fig. 3G), and each sample type and age in isolation (Fig. 3H). Significant differences (*P* < 0.05 via PERMANOVA; data tables in Fig. 3G and H) were observed for all variables examined (genotype, sex, sample type, cohort [age], and cage assignment), with the exception of genotype for the 4-month-old cecal and fecal samples. In all cases examined, the cage resulted in the largest proportion of variance explained (PERMANOVA R^2^ > 50%). The proportion of variance explained by genotype showed a remarkable increase as the animals aged, indicating a gradual divergence in microbiome composition between the WT and 5xfAD animals (Fig. 3H, inset). For both the 18-month-old cecal and fecal samples, nearly 10% of the variance was explained by genotype. Age also accounted for a substantial portion of the variance (R^2^ > 16% in all cases). Sex and genotype accounted for more of the variance in the cecal samples than in the fecal samples. The full PERAMANOVA statistics are provided in Table S1. An important point to note about this analysis is that many of the variables used in this comparison are inextricably conflated as: (i) littermates of the same sex were often (but not always) housed together; (ii) males and females were always housed separately; and (iii) mice of different age groups were housed separately. Because littermates were housed together, no effort was made to disentangle cage effects from maternal effects.

### Microbial species whose abundances differed between 5xfAD and WT animals.

**FIG 4:**
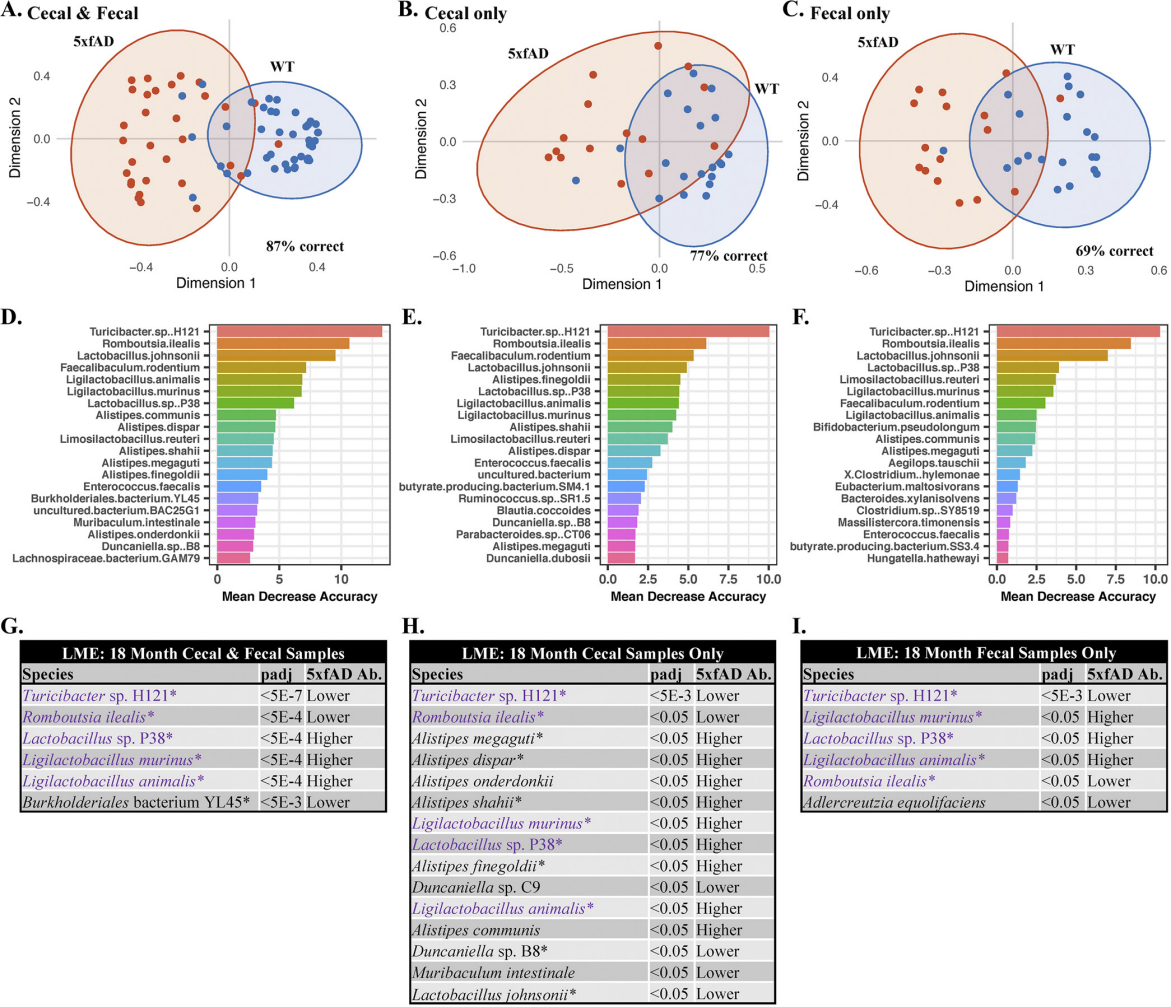
Random forest and linear mixed effects (LME) modeling of microbiomes from 18-month-old animals. All evaluations were performed with separation by genotype (WT versus 5xfAD). Random forest (A–F) of the cecal and fecal samples combined (A and D), the cecal samples only (B and E), and the fecal samples only (C and F). *Turicibacter* sp. H121 was responsible for the largest proportion of separation in all of the random forest analyses, with a mean decreased accuracy from 10 to 14%. LME (G–I) was performed with Housing ID as the random variable. Shown are the cecal and fecal samples combined (G), the cecal samples only (H), and the fecal samples only (I). The “5xfAD Ab.” column in panels G–I indicates whether the species was found in relatively higher or lower abundance in the 5xfAD samples, compared to the WT samples. The species in purple were found to be significantly different in all three comparisons, and species with an asterisk were among the top drivers of separation in the random forest analysis. The LME computed *P* values (*P* adj) are false-discovery rate (FDR) corrected.

Random forest was used to elucidate specific species-level differences in the microbiome between genotypes (Fig. 4A–F). AD pathology became more pronounced as the mice age. Therefore, the 18-month-old mice were the primary focus of this analysis. Clear separation was observed between genotypes, both when data from the cecal and fecal sample types were combined (Fig. 4A) and when they were considered separately (Fig. 4B and C). In all three cases, overlapping ellipses (which represent the 95% confidence intervals) showed that the groups share many features in common. *Turicibacter* sp. H121 was the species most responsible for the random forest separation (>10% mean decreased accuracy in all separations), with Romboutsia ilealis and Lactobacillus johnsonii also playing substantial roles (Fig. 4D–F).

Random forest of the 18-month-old samples with grouping by sample type and sex (ignoring genotype differences) also showed interesting trends (Fig. S2A–D). Grouping by sample type showed an out-of-bag accuracy of 90%, with *Oscillibacter* sp. PEA192, Flavonifractor plautii, and Desulfovibrio desulfuricans contributing heavily to the separation (Fig. SA and B2). Grouping by sex was 97% accurate, with *Limosilactobacillus reuteri* and Lactobacillus johnsonii playing the largest role in the separation (Fig. S2C and D). Differences with respect to age were also examined (Fig. S2E and F), with large overlap between each age cohort. The 4-month samples showed the most variation and the least overlap relative to the other age cohorts. Staphylococcus nepalensis was the species most responsible for the separation by age, with a mean decreased accuracy (MDA) of almost 12%.

10.1128/mbio.01794-22.3 FIG S2 Random forest analysis. (A and B) 18-month samples with grouping by sample type. (C and D). 18-month samples with grouping by sex. (E and F) All ages with grouping by age. Download FIG S2, PDF file, 0.1 MB. Copyright © 2022 Dunham et al. 2022 Dunham et al. https://creativecommons.org/licenses/by/4.0/ This content is distributed under the terms of the Creative Commons Attribution 4.0 International license.

Random forest is generally applied as a classification method and can lead to false positives when used to identify differentially abundant species. Therefore, we also applied a linear mixed-effects (LME) model to identify bacterial species whose abundances significantly differed between the WT and 5xfAD genotypes (Fig. 4G–I). Using housing ID as the random effect, LME modeling was performed on all samples in aggregate (i.e., 5xfAD [all age groups & sample types] versus WT [all age groups & sample types]) as well as to each age group and sample type individually. The comparisons made for the 18-month samples were: 5xfAD fecal and cecal versus WT cecal and fecal (Fig. 4G), 5xfAD cecal versus WT cecal (Fig. 4H), and 5xfAD fecal versus WT fecal (Fig. 4I). After correcting for the false discovery rate, significantly different species were only found in the 18-month samples. The 18-month cecal samples had 15 differentially abundant species (*P* adj < 0.05), whereas the other two groupings each had six differentially abundant species. Five species were significant across all three groupings: *Turicibacter* sp. H121, Romboutsia ilealis, *Lactobacillus* sp. P38, Ligilactobacillus murinus, and Ligilactobacillus animalis. Among others, six species from the genus *Alistipes* were differentially abundant in the 18-month cecal samples only. A large proportion of the species (11/17) were also among the top 20 species contributing to the random forest separations.

Relative abundance plots for all 17 differentially abundant species can be found in Fig. S3, with faceting by sample type and sex to allow for the inspection of more subtle abundance differences. The abundances of 8 of the 17 significant species were lower in the 18-month-old 5xfAD mice, and 9 of the 17 species were present at a higher abundance. With the exceptions of *Ligilactobacillus murinus* (relative abundances as high as 50%), Lactobacillus johnsonii (relative abundance as high as 30%), and Muribaculum intestinale (relative abundance as high as 16%), the differentially abundant species were minor contributors to the overall microbiome composition, displaying relative abundances below 5%.

10.1128/mbio.01794-22.4 FIG S3 Relative abundance plots for the 17 species with significantly different abundances (*P* adj < 0.05) via all three LME comparisons. The plots are faceted by sample type and sex. Species that were significantly different within each facet are denoted with an asterisk (*, *P* < 0.05 and **, *P* < 0.005 via a Wilcoxon signed rank test). The box plots show the median relative abundance +/− the first and third quartiles, with the whiskers showing the range or 1.5× the interquartile range, whichever was less. Download FIG S3, PDF file, 0.6 MB. Copyright © 2022 Dunham et al. 2022 Dunham et al. https://creativecommons.org/licenses/by/4.0/ This content is distributed under the terms of the Creative Commons Attribution 4.0 International license.

### Multiple *Turicibacter* species were significantly less abundant in the 18-month-old 5xfAD animals.

**FIG 5:**
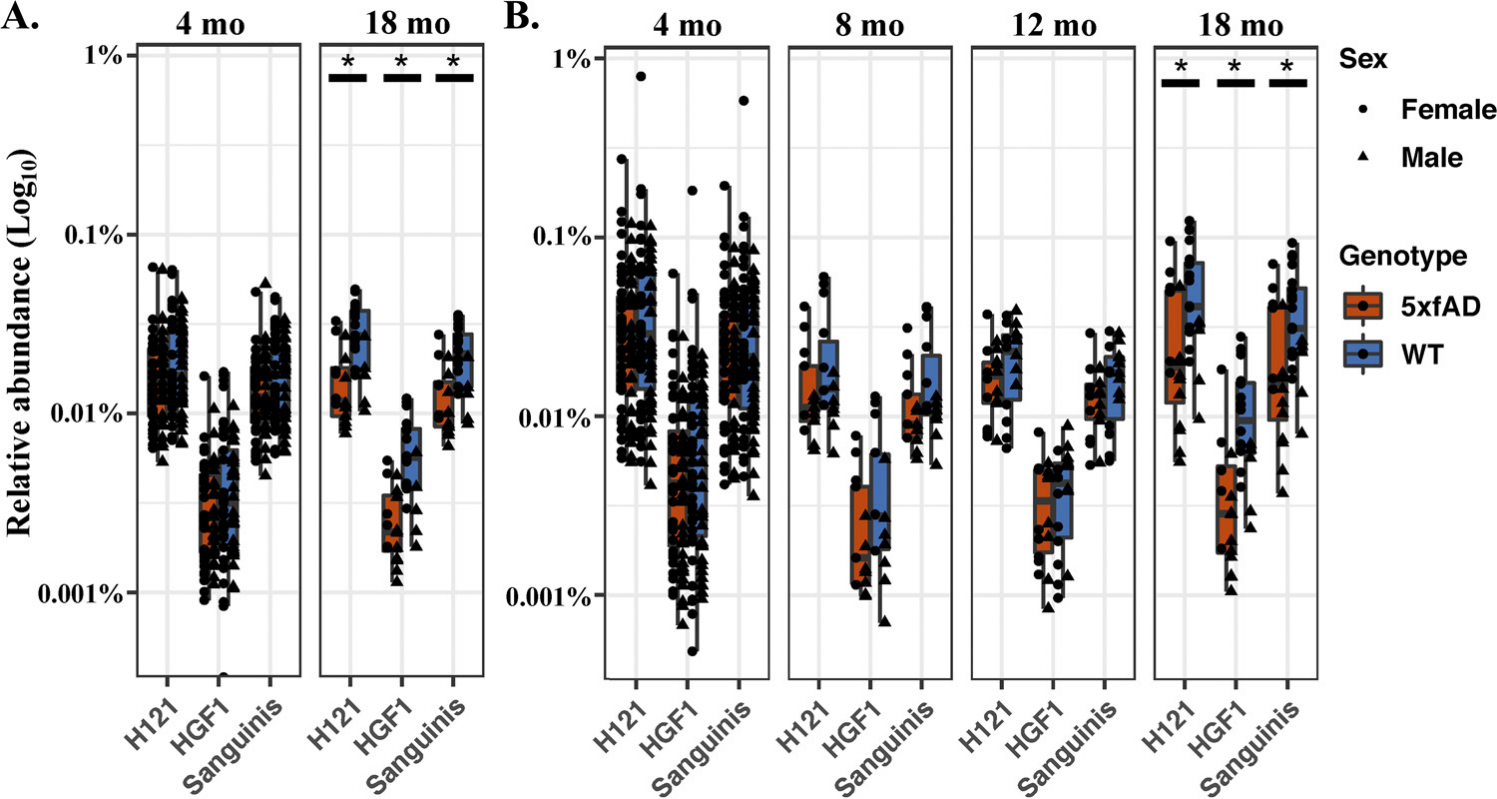
Relative abundances for *Turicibacter* spp. in the cecal (A) and fecal (B) samples generated via Bowtie2. Shown are *T. sanguinis*, *T*. sp. H121, and *T*. sp. HGF1. The WT and 5xfAD cohorts in panels A and B are significantly different (*P* < 0.05 via a Wilcoxon rank sum test) for all three *Turicibacter* species at 18 months of age only.

*Turicibacter* was recently identified as a potential mediator of the gut-brain axis in AD through its ability to consume and regulate the production of, 5-hydroxytryptamine (5-HT, serotonin) (33). Further studies have highlighted the potential role of serotonin in AD (recently reviewed by Aaldijk et al. in 2022) (34). These established findings, as well as the importance of *Turicibacter* sp. H121 in our random forest and LME analysis, motivated us to pursue a more detailed investigation. A Bowtie2 sequence alignment was performed against reference genomes for three species of *Turicibacter* (*T.* sp. H121, *T.* sp. HGF1, and T. sanguinis) that encode a neurotransmitter sodium symporter-related protein with sequence and structural homology to the mammalian serotonin transporter (SERT; see Materials and Methods for more details). All three species were present in both the fecal and cecal microbiomes (Fig. 5A and B), and, in agreement with the NCBI BLAST data for *T.* sp. H121 shown in Fig. S3, they were significantly less abundant in the 5xfAD mice, relative to the WT animals at 18 months of age (*P* < 0.05). Any apparent differences at other ages were not statistically significant.

### Plasma metabolomics reveals differentially abundant phospholipids and amino acids with respect to age, sex, and genotype.

Quantitative metabolomics was performed on the blood plasma of the 12- and 18-month-old mice in our study. For these data, we utilized the Biocrates Absolute-IDQ P180 Kit (see Materials and Methods), which was previously applied to develop blood-based biomarker assays for preclinical AD (35–37). From this list of 188 potential analytes, we received concentrations for 137 metabolites within the assay’s quantitation limits for more than 50% of our samples, which was the quality threshold used for this report.

The metabolites showed similar aggregate concentrations in the 12 and 18 month samples (*P* > 0.05 via a Kruskal-Wallis test) (Fig. S4A). The Shannon alpha diversity was also similar across all age and genotype groupings (*P* > 0.05 via a Kruskal-Wallis test) (Fig. S4B). PCoA did not reveal clear separation with respect to age, sex, or genotype (Fig. S4C). PERMANOVA of the Bray-Curtis dissimilarity matrix (Fig. S4D) showed sex to be the only significant distinguishing factor (*P* = 0.037; R^2^ = 0.069). Housing ID accounted for almost 40% of the variance in the data set, but it was not significant (*P* = 0.357; R^2^ = 0.39).

10.1128/mbio.01794-22.5 FIG S4 Alpha and beta diversity metrics for plasma metabolomics. (A) Summed concentrations for all metabolites. (B) Shannon diversity. (C) PCoA. (D) PERMANOVA of the Bray-Curtis dissimilarity matrix. Download FIG S4, PDF file, 0.10 MB. Copyright © 2022 Dunham et al. 2022 Dunham et al. https://creativecommons.org/licenses/by/4.0/ This content is distributed under the terms of the Creative Commons Attribution 4.0 International license.

**FIG 6:**
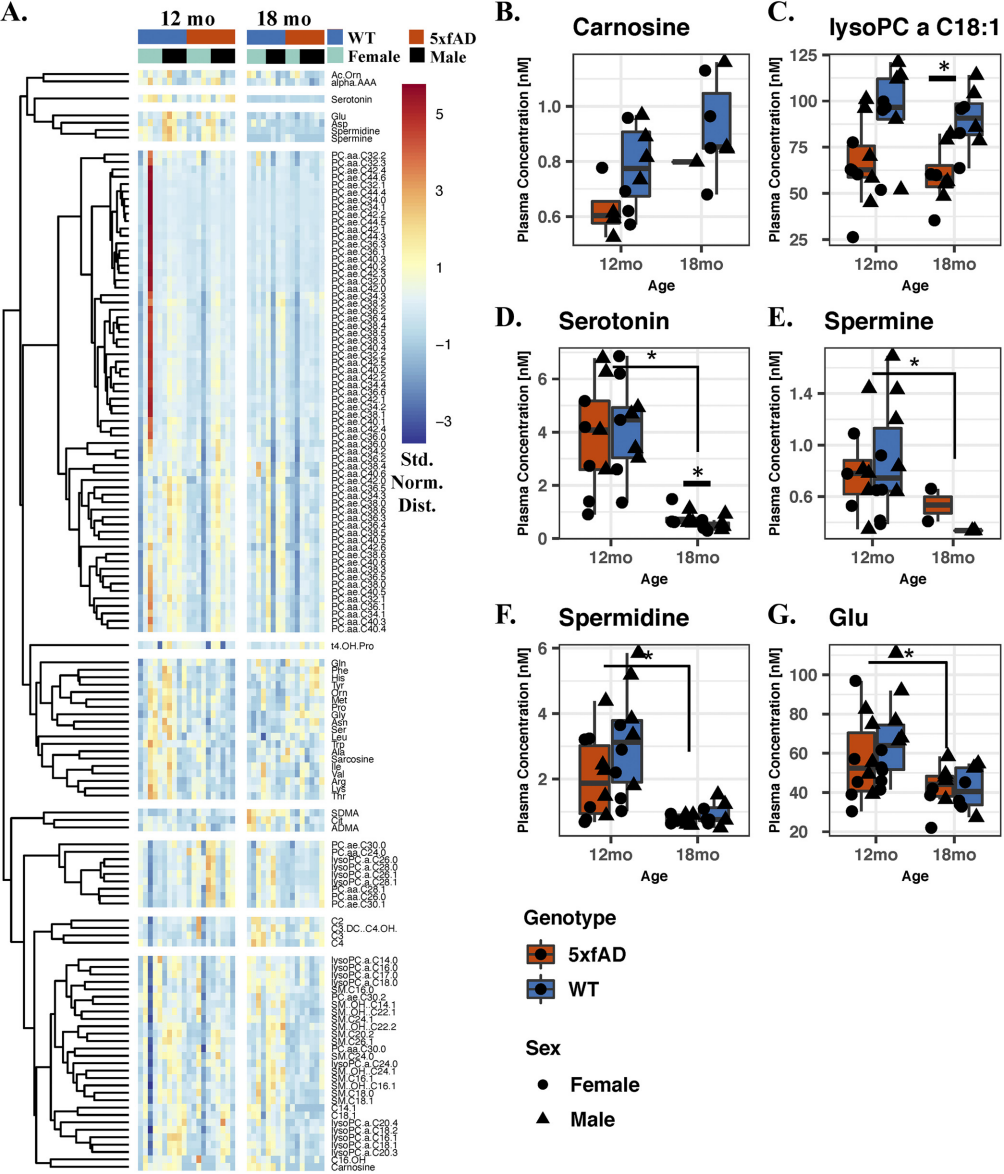
Quantitative plasma metabolomics. (A) Heat map of the 137 quantified metabolites in the plasma of 12- and 18-month-old mice. Shown is the standard normal abundance distribution, with hierarchical clustering to group metabolites with similar abundance trends. Metabolites with significantly different concentrations with respect to genotype (*P* adj < 0.05 via LME) (B and C). Metabolites with significantly different concentrations with respect to age (*P* adj < 0.05 via LME) (D–G). The asterisks in panels B–G indicate the metabolites that were found to significantly differ in each comparison via a Wilcoxon signed rank test (*P* < 0.05).

A clustered heat map of all 137 quantified plasma metabolites (Fig. 6A) shows metabolite specific differences with respect to age, genotype, and sex. Relative to their 12-month-old counterparts, the 18-month-old mice appeared to have lower concentrations of α-aminoadipic acid, serotonin, glutamic acid, aspartic acid, spermidine, and spermine. Most of the detected phosphatidylcholines (PCs) were depleted in the 18-month-old 5xfAD mice (both diacyl [aa] and acyl-alkyl [ae] PCs), while many amino acids (shown primarily in the sixth cluster) had an increased relative concentration. Sex-specific differences were pronounced for most of the detected PCs, which clustered together in the fourth cluster.

Random forest (Fig. S5) showed separation by age (75% out-of-bag accuracy), sex (78% accurate), and genotype (81% accurate for the 18-month samples only). Serotonin was the metabolite most responsible for age-related separation (MDA = 8.5%), closely followed by spermidine (MDA = 7%) and spermine (MDA = 7%). Separation with respect to sex was dominated by sphingomyelin sphingolipids (SMs) and phosphatidylcholines (PCs) from both the diacyl (aa) and acyl-alkyl (ae) subclasses. SM C20:2, PC aa C40:3, PC aa C42:6, PC ae C42:0, PC aa C34:3, PC ae C34:2, and SM (OH) C22:2 topped this list, with each accounting for more than 5% of the MDA. Random forest separation with respect to genotype was negligible when both of the age groups were considered together (61% accurate). The most predictive molecules for genotype at 18 months of age were glycine (MDA = 7%), carnosine (MDA = 4.5%), serine (MDA = 4.5), SM C24:1 (MDA = 4.2%), and serotonin (MDA = 4%).

10.1128/mbio.01794-22.6 FIG S5 Random forest analysis of the plasma metabolome. (A) 12-month and 18-month samples by age. (B) 12 and 18-month samples by sex. (C) 18 month samples only by genotype. Download FIG S5, PDF file, 0.1 MB. Copyright © 2022 Dunham et al. 2022 Dunham et al. https://creativecommons.org/licenses/by/4.0/ This content is distributed under the terms of the Creative Commons Attribution 4.0 International license.

LME was also performed, using the housing ID as the random effect, and several significantly different metabolites (*P* adj < 0.05) were observed with respect to age, sex, and genotype (Fig. S6A). The concentrations of the metabolites that significantly differed between genotypes are shown in Fig. 6B and C, between ages in Fig. 6D–G, and between sexes in Fig. S6B. Carnosine and lysoPC a C18:1 had significantly lower plasma concentrations in the 5xfAD mice, relative to those of the WT mice (*P* adj < 0.05). Serotonin, spermine, spermidine, and PC aa C36:0 each had significantly lower concentrations in the 18-month-old mice, relative to the younger cohort. The concentrations of two SM lipids, two PC ae lipids, six PC aa lipids, and lysine significantly differed between sexes. Separately, a targeted analysis using the Wilcoxon signed rank test revealed serotonin to be more concentrated in the 18-month-old 5xfAD animals, relative to their age-matched counterparts (*P* = 0.038) (Fig. S7A).

10.1128/mbio.01794-22.7 FIG S6 Significant plasma metabolites via LME. (A) Comparisons between metabolomes of 12 and 18-month-old WT and 5XFAD animals. (B) Significantly different plasma metabolites with respect to sex. Download FIG S6, PDF file, 0.2 MB. Copyright © 2022 Dunham et al. 2022 Dunham et al. https://creativecommons.org/licenses/by/4.0/ This content is distributed under the terms of the Creative Commons Attribution 4.0 International license.

10.1128/mbio.01794-22.8 FIG S7 Selected metabolites in plasma and feces. (A) Concentration of serotonin in plasma from 18-month-old 5xfAD animals. Serotonin is more concentrated in the 18-month-old 5xfAD animals, relative to their age-matched counterparts (*P* < 0.05 via a Wilcoxon signed rank test). (B) Normalized abundance of tryptophan in fecal samples. Differences by genotype are not significant (*P* > 0.05 via a Wilcoxon signed rank test). (C) 5-hydroxyindoleacetic acid (5-HIAA) in the fecal samples. 5-HIAA is differentially abundant when comparing WT and 5xfAD samples at 8 and 12 months of age (*P* < 0.05 via a Wilcoxon signed rank test). Download FIG S7, PDF file, 0.1 MB. Copyright © 2022 Dunham et al. 2022 Dunham et al. https://creativecommons.org/licenses/by/4.0/ This content is distributed under the terms of the Creative Commons Attribution 4.0 International license.

### Correlations between the microbiome and the plasma metabolome.

**FIG 7:**
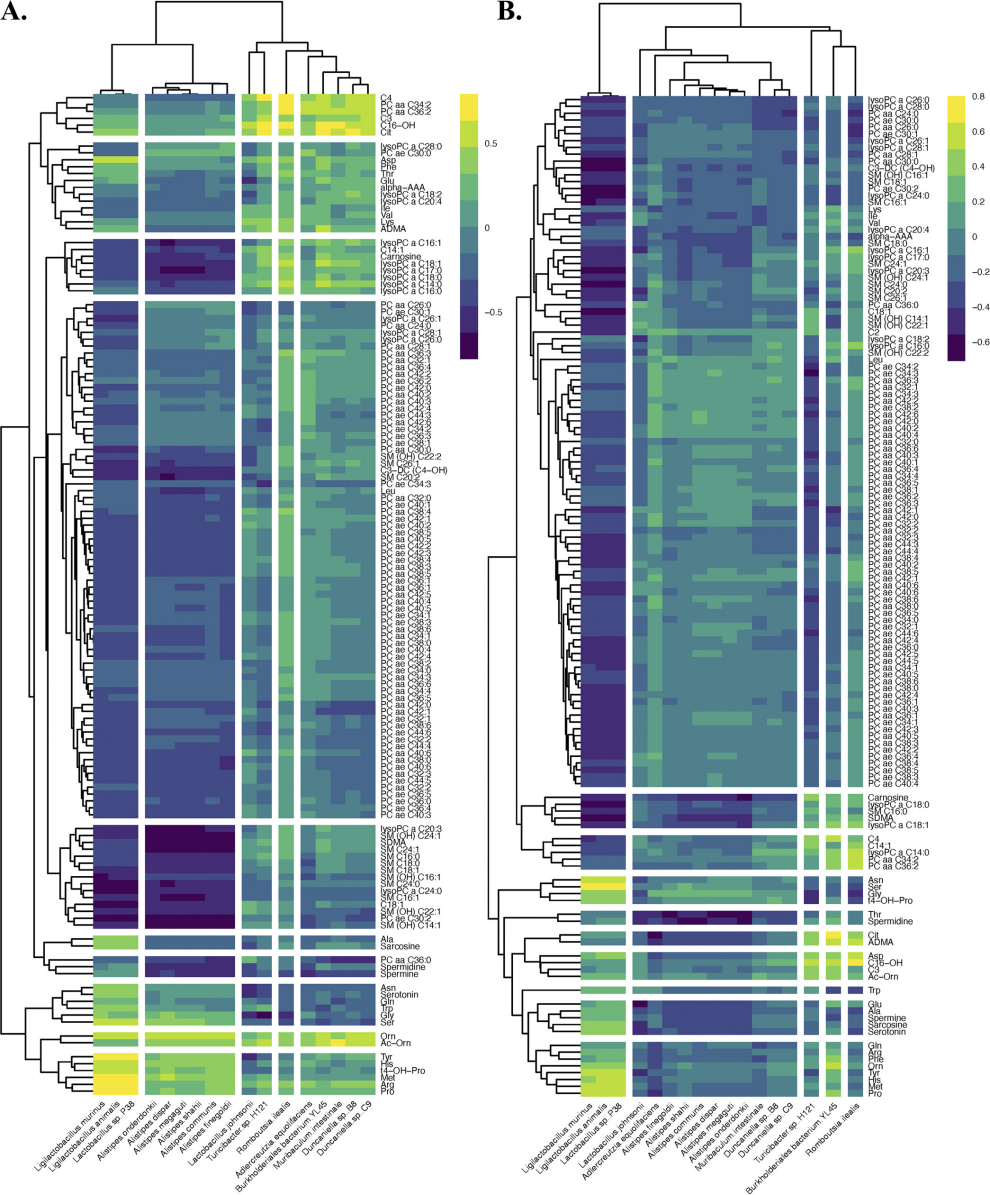
Spearman correlations between the 17 significant microbial species and all 137 quantified plasma metabolites at 18 months of age in the (A) cecal and (B) fecal samples.

Spearman correlations were generated to better evaluate the relationships between the significant microbes at 18 months of age and the plasma metabolome. The Spearman correlations between all 137 quantified plasma metabolites and the 17 significant microbes for the 18-month-old mice are provided in Fig. 7. The analogous correlations for all 137 plasma metabolites and the 100 most abundant microbes are provided in Fig. S8A and S8B.

10.1128/mbio.01794-22.9 FIG S8 Spearman correlation between all 137 quantified plasma metabolites and the 100 most abundant microbes in the (A) cecal and (B) fecal samples. Download FIG S8, PDF file, 0.2 MB. Copyright © 2022 Dunham et al. 2022 Dunham et al. https://creativecommons.org/licenses/by/4.0/ This content is distributed under the terms of the Creative Commons Attribution 4.0 International license.

For the cecal samples (Fig. 7A), strong correlations were observed between the first cluster of bacteria (*Ligilactobacillus murinus*, *Ligilactobacillus animalis*, and *Lactobacillus* sp. P38) and PC ae C30:0, ornithine, acetylornithine, tyrosine, histidine, methionine, arginine, and proline (among others). These bacteria were anticorrelated with most lipids. Similarly, the six *Alistipes* species (which were found together in the second microbial cluster) weakly co-occurred with several amino acids in the last two metabolite groupings. The remaining 8 microbes (which were all at reduced relative abundances in the 18-month-old 5xfAD samples) showed correlation trends similar to one another. They were weakly anticorrelated with most of the metabolites, aside from three acylcarnitines (propionylcarnitine [C3], butyrylcarnitine [C4], exadecenoylcarnitine [C16-OH]), PC aa C34:2, PC aa C36:2, citrulline, and acetylornithine.

All 17 microbes in the fecal correlation (Fig. 7B) were either anticorrelated or weakly correlated with the top group of 102 metabolites. *Ligilactobacillus murinus*, *Ligilactobacillus animalis*, and *Lactobacillus* sp. P38 were correlated with most of the amino acids and many of the acylcarnitines, and they were strongly anticorrelated with most of the lipids. The six *Alistipes* species, Muribaculum intestinale, *Duncaniella* sp. B8 and *Duncaniella* sp. C9 were weakly correlated with or anticorrelated with all 137 metabolites. *Turicibacter* sp. H121 and Romboutsia ilealis were weakly correlated with many amino acids, acylcarnitines, and biogenic amines, and they differed from *Burkholderiales* bacterium YL45, based primarily on the last cluster of 8 amino acids.

Untargeted metabolomics (see Materials and Methods for more details) were also performed on a subset of fecal samples with adequate material, namely, 40 samples from 4-, 8-, and 12-month-old mice. Unfortunately, there was insufficient material to perform both metabolomics and genomics for the 18-month-old mice and therefore there are no fecal metabolomics data for this age cohort. Overall trends did not reveal any major differences with respect to genotype. Fig. S9 contains a more thorough review of this data set, including the normalized abundances of the six fecal metabolites that were found to be significantly different in the 12-month-old females by LME (Fig. S9A–F) and the Spearman correlations between the 100 most abundant fecal metabolites and 17 significant species at 18 months of age (Fig. S9G).

10.1128/mbio.01794-22.10 FIG S9 Analysis of the fecal metabolome (*n* = 40 samples from 4-, 8-, and 12-month-old mice). The relative abundances were produced for 684 named metabolites with various degrees of identification confidence, as well as for more than 3,500 unidentifiable ions, which were not considered here. A PERMANOVA with genotype, sex, age, and housing ID revealed age to be the only significant parameter (*P* = 0.013). Age accounted for approximately 9% of the total variance. The housing ID matched the 50% variance observed in the microbiome; however, this parameter was not significant (*P* = 0.34). (A–F) The normalized abundances of the six fecal metabolites were found to be significantly different at 12 months of age (females only) via LME (*P* adj < 0.05). All significant metabolites (nialamide, fluorene, the tripeptide Ala-Pro-Lys, ferulic acid, 4 acetylbutyric acid, and disaccharide 16) were more abundant in 12-month-old 5xfAD female samples, relative to the age- and sex-matched controls. No significant metabolites were found with the other WT versus 5xfAD comparisons. (G) Spearman correlation between the 100 most abundant metabolites and 17 significant species at 18 months of age (as revealed by the microbiome LME analysis). Distinct patterns of coexpressed microbes and bacteria can be seen, with a Spearman’s co-occurrence ranging from −0.7 to 0.5. Romboutsia ilealis and *Turicibacter* sp. H121 were weakly associated with most metabolites and did not show pronounced correlation patterns. Download FIG S9, PDF file, 0.1 MB. Copyright © 2022 Dunham et al. 2022 Dunham et al. https://creativecommons.org/licenses/by/4.0/ This content is distributed under the terms of the Creative Commons Attribution 4.0 International license.

## DISCUSSION

### Overview of microbiome composition and changes.

Overall, the richness and Shannon diversity of the fecal microbiomes increased between 4 and 8 months, and they declined thereafter (Fig. 3A and B). Among other factors, changes in gut microbial diversity have been associated with age (38), sex (38), obesity (39), and general health (40). PERMANOVA (Fig. 3G and H) revealed a marked age-associated increase in microbiome variation attributable to genotype (Fig. 3H), rising from 0.9 to 9% for the cecal samples, and from 0.3 to 8% for the fecal samples. This trend indicates that the 5xfAD and WT microbiomes increasingly diverge as the animals age and that the AD pathophysiology becomes more pronounced and chronic. Most of the variance in the fecal and cecal microbiomes was attributable to the specific cages in which the animals resided, a finding that is consistent with the results from other studies of fecal microbiomes in 5xfAD mice (21, 30). Such “cage effects” are routinely observed in microbiome studies, and they serve to increase variability and complicate the associations between trends in the microbial population and the experimental factors of interest (41–44). The consistency of these findings emphasizes the need for the thorough documentation of housing protocols and the accounting for cage effects in the experimental design. Cage effects have been covered extensively in the microbiome literature, and these and other experimental pitfalls were expertly discussed by Kim et al. (45).

At first approximation, housing animals from the control and experimental cohorts together (as we did here) helps the researcher disentangle cage effects from genotype differences; however, this may obscure the true extent of microbiome dysbiosis. For example, in their study of the effects of sodium oligomannate on 5xfAD mice (some of which were housed together with WT controls and others separate), Wang and coworkers found that the WT microbiomes more closely resembled those from cohoused 5xfAD animals than those from WT controls housed separately. Remarkably, Alzheimer’s-associated disease hallmarks, including those both behavioral (e.g., decline in discrimination learning) and physical (e.g., decreased development of M1-type glial cells), also converged with cohousing. This convergence was attributed to coprophagy-induced microbiome dysbiosis of the WT animals (21). Another option is to house each animal in its own cage (suggested by Kim et al. and others) (45); however, this may be prohibitively expensive or even lead to the unintended distortion of results due to social isolation.

### Differentially abundant species.

Comparing the microbiome composition of the 5xfAD mice to that of the WT B6J controls revealed differences later in life (Fig. 4). The abundances of 17 species of bacteria were found via LME to be significantly different between genotypes at 18 months of age (15 species in the cecal samples and 6 in the fecal samples) (Fig. 4G–I). Of the 17 significant species, 8 were found at reduced abundances in the 5xfAD mice, relative to the WT controls (*Turicibacter* sp. H121, Romboutsia ilealis, *Burkholderiales* bacterium YL45, Adlercreutzia equolifaciens, *Duncaniella* sp. PC9, *Duncaniella* sp. B8, Muribaculum intestinale, and Lactobacillus johnsonii), and 9 were found at increased abundances (*Lactobacillus* sp. P38, *Ligilactobacillus murinus*, *Ligilactobacillus animalis*, and 6 *Alistipes* species). A closer look at the random forest analysis (Fig. 4A–F; Fig. S2) shows some concordance between the top variables of importance that are responsible for the separation by genotype and sex, including *Turicibacter* sp. H121 and Lactobacillus johnsonii. This agreement may be the reflection of a sex-specific difference in AD-pathology manifesting as differential microbial dysbiosis.

Our findings are largely consistent with those of previous studies of the fecal microbiome of people with AD. Using 16S rRNA sequencing, Vogt et al. reported significantly decreased levels of 7 genera of bacteria in people with AD, including *Turicibacter* and *Adlercreutzia*. They also reported 7 genera with increased abundances in people with AD, including *Alistipes* (25). Considering the vast differences in physiology, diet, and environment, the concordance of our findings in 5xfAD mice with those in people with AD is remarkable, and may suggest AD related differences in the gut microbiome that are independent of the host.

### *Turicibacter* spp. and serotonin.

The finding of reduced *Turicibacter* spp. is also noteworthy because members of this genus can both metabolize and induce the production of serotonin in the gut. The association between serotonin and *T. sanguinis* has been shown to be both causative and bidirectional (46). Along with depleted *Turicibacter* (25), both blood-derived and central nervous system-derived serotonin are depleted in humans with AD pathology (34, 47, 48).

Here, we found three *Turicibacter* spp. (i.e., *T.* sp. H121, *T.* sp. HGF1, and *T. sanguinis*) to be significantly less abundant in 5xfAD mice (relative to WT mice) at 18 months of age (Fig. 5).

Our measurement of serotonin in the blood plasma of 12- and 18-month-old mice showed the neurotransmitter to be at a significantly higher concentration in 18-month-old 5xfAD animals relative to age-matched WT controls (Fig. 6D; Fig. S7A). Although this finding is surprising due to the difference between our serotonin results and corresponding measurements in human plasma, it has been reported by others for 5xfAD mice (21). Because of the potential importance of serotonin to AD, it must be investigated whether this discrepancy between patients with AD and 5xfAD mice extends to other animal models. The other remarkable finding with regard to serotonin was the dramatic drop in concentration between 12 and 18 months of age, which may have larger implications for the health and experimental validity of the older mice. Alternatively, the drop in serotonin levels may be related to dynamic interactions between chronic, established AD pathology and associated nonlinear physiological responses.

Serotonin was not detected in our fecal metabolomics data; however, we did detect tryptophan (which is the primary biochemical precursor of serotonin synthesis) as well as one of its bioactive metabolites (5-hydroxyindoleacetic acid; 5-HIAA) (Fig. S7B and C). 5-HIAA was significantly more abundant in the fecal material of 5xfAD mice, relative to their WT littermates at both 8 and 12 months of age, whereas the differences in tryptophan abundance were not significant. This finding may indicate the increased synthesis and metabolism of serotonin in the gastrointestinal tracts of 5xfAD mice, which in turn results in higher levels of its primary metabolite 5-HIAA.

It is important to note that the relationship between gut, blood, and brain serotonin is complicated and that causative associations are difficult to establish. For example, although more than 90% of the body’s serotonin is made in the gut, it does not cross the blood-brain barrier (48).

### Plasma metabolomics.

The two plasma metabolites found via LME to be significantly lower in concentration in 18-month-old 5xfAD animals (carnosine and lysoPC a C18:1) (Fig. 6B and C) have both been associated with AD. Among other positive effects (49), carnosine (a dipeptide comprised of beta-alanine and histidine) and related compounds have been found to suppress beta-amyloid toxicity (50), suggesting that lower concentrations in the bloodstream may exacerbate AD symptoms. Interestingly, lysoPC a C18:2 (differing from lysoPC a C18:1 only by the position of the double bond) was previously found by Fiandaca et al. to be lower in people who were at risk for developing AD (36). Several other P180 biomarkers highlighted by both Fiandaca et al. (36) and Mapstone et al. (35) showed trends in our data that were similar to those in people with AD. However, following quality control, they lacked adequate statistical power with which to provide meaningful context to this report.

### Limitations and weaknesses.

The dominance of cage effects undoubtedly obscures other interesting trends in the microbiome and metabolome, including differences due to sex, age, and genotype. The cage effects were also inexorably linked to other factors, including maternal identity, as same-sex littermates were housed together. This linkage may cause even more pronounced cage effects, contributing to the large proportion of variance attributed to the housing ID. Interestingly, the proportion of variance attributable to housing was approximately equivalent for both the fecal and the cecal samples (Fig. 3G and H; Table S1). In our opinion, the housing of AD model animals needs careful and systematic study, not only from the perspective of the microbiome and the metabolome but also with the consideration of microbiome-induced changes in behavior and neuropathology. Further innovation in animal husbandry strategies may also be warranted (45, 51).

Our results from plasma metabolomics, although powerful, were limited by many measurements falling outside the quantitative range. This resulted in the necessary exclusion of many metabolites and the deterioration of statistical power. Future experiments should strive to overcome this issue by using a larger sample volume or perhaps a more sensitive metabolomics method. Depending on the question at hand, earlier time points (e.g., 4 months) could also be informative.

Unfortunately, we had inadequate material to perform both genomics and metabolomics on all fecal samples, forcing us to choose between the two techniques in some cases, including at the critical time point of 18 months. We prioritized genomics, and, as a result, we were only able to collect metabolomics for a subset of the samples (namely, the 4-, 8-, and 12-month fecal samples). Due to the advanced stage of AD pathology, it is likely that an analysis of the 18-month metabolomes would have produced more meaningful results than the analysis with the younger animals.

Finally, in this work, we largely confined our analysis of the microbiome to the perspective of taxonomy. Evaluating this same data from the standpoint of functional genomics, that is, how functionally predicted genes identified through metagenomics correlate with detected metabolites in the blood or feces, may yield additional insights.

## CONCLUSION

Here, we describe the longitudinal gut microbiome and metabolome of a 5xfAD murine model for familial AD. We found several species of bacteria that were both overexpressed and underexpressed in the AD mice, relative to their WT counterparts, many of which matched previous observations of the fecal microbiome in persons with AD (25). In line with the findings of previous studies (21, 30), we also found a large amount of variance in the microbiome due to housing assignment. Future studies must account for cage effects in their experimental designs, as even with meticulous animal husbandry, cage effects are likely to be the largest source of variance in the murine microbiome. In our case, we were able to confidently compare differences arising due to genotype among age-matched littermates of the same sex that were housed together.

One key finding was that three species of *Turicibacter* were present at lower abundances in the 18-month-old 5xfAD mice, relative to the age-matched WT controls. Some species of *Turicibacter* are known to both metabolize and regulate the production of serotonin (33); however, the serotonin concentrations were found to be significantly elevated in the 18-month-old 5xfAD mice, relative to the age-matched controls. Serotonin was also observed to be nearly 10-fold less concentrated in the blood plasma of the 18-month-old mice, relative to that observed in the 12-month-old cohort. Depending on the importance of serotonin in AD, this finding may have broad implications for the design of better murine models and for our understanding of the role of the microbiome in the etiology of AD.

This research adds to our collective understanding of the microbiome gut-brain axis in neurodegeneration and serves as a foundation for further microbiome research with AD mouse models. Our findings motivate further studies involving gnotobiotic and wildling mice, probiotics, and diet interventions, among other investigations.

## MATERIALS AND METHODS

### Animal conditions and fecal collection.

All of the animal experiments were approved by the UC Irvine Institutional Animal Care and Use Committee, and they were conducted in compliance with all of the relevant ethical regulations for animal testing and research. The animals were bred and aged together in the Transgenic Mouse Facility at UCI, and they were maintained in a 12/12-h light/dark cycle. 5xFAD hemizygous (B6.Cg Tg(APPSwFlLon,PSEN1*M146L*L286V)6799Vas/Mmjax, Stock number 34848-JAX, MMRRC) and its wild-type littermates were produced via crossing or IVF procedures with C57BL/6 J (Jackson Laboratory, ME) females. After weaning, they were housed together with same-sex littermates until harvest. Littermate female and male mice from 4, 8, 12, and 18 months of age were used in this study. All of the mice were fed a LabDiet Irr6f (7% fat) diet and were sustained on pH 2.5 to 3.0 autoclaved water. Samples were collected at 4, 8, 12, and 18 months of age, based on multiple factors, including the known progression of AD pathology, interest in dynamic accumulation, long-term exposure to AD pathology, especially amyloid plaques, and synergy with other ongoing studies of the same animals.

Immediately prior to sampling or sacrifice, the animals were isolated into individual cages for fecal collection. All working areas and tools were sterilized with 70% ethanol. Fecal pellets and cecum samples were deposited directly into 1.5 mL Eppendorf tubes. All sample tubes were placed on dry ice following collection. All samples were stored at −80°C until analysis.

### Sequence library preparation.

DNA was extracted from both fecal and cecal samples by Zymo Research Corp. using the ZymoBIOMICS-96 MagBead DNA Kit (Zymo Research, Cat. number D4302). The quantity of DNA in each sample was measured with a Quant-iT PicoGreen dsDNA Assay Kit (ThermoFisher, Cat. number P11496) and read with a Synergy H1 Microplate Reader (BioTek, Cat. number BTH1M). The sequence libraries were prepared from the extracted DNA samples using a Nextera DNA Flex Library Prep Kit (Illumina, Cat. number 20018705), and a low volume variation of the standard protocol was followed (52).

The average input DNA for each sample was 391.6 ng (std dev = 54.8 ng). Samples were prepared for PCR with Kapa HiFi HotStart ReadyMix (Roche, Cat. number 07958935001) using the following primers: KAPA-PCR-F: 5′–AATGATACGGCGACCACCG*A−3′ and KAPA-PCR-R: 5′–CAAGCAGAAGACGGCATACG*A–3′.

PCR was performed with an Eppendorf Mastercycler Nexus Gradient (Eppendorf, Cat. number 2231000665), using the standard thermal cycles for the Nextera Flex Kit. The resulting sequence fragments were analyzed on an Agilent Bioanalyzer to determine the fragment length distribution (Agilent, Cat. number G2939BA). The sequence libraries were pooled based on DNA concentration, as determined by the Bioanalyzer. After pooling, the libraries were sent to Novogene Co., Ltd. for sequencing on an Illumina HiSeq4000.

### Sequence data processing.

The sequence libraries were downloaded from the Novogene FTP website. The libraries consisted of 456 samples pooled into a single dual-index, paired-end library and run across two lanes. The two paired-end aggregate FASTQ files were parsed using BBDuk to trim adapters and remove artifact mouse DNA (GCA_000001635.8), and rat DNA (GCA_000001895.4). The cleaned files were demultiplexed using demuxbyname.sh inside the BBmap suite. To assign microbial taxonomy, GenBank’s NT database (version 1/29/20) was retrieved using the update_blastdb PERL script. Each pair of metagenome files was queried against NT using GenBank’s MEGABLAST algorithm from the BLAST+ package (version 2.10.0), using all default parameters. For the phylogenic assignment, the NCBI Taxonomy database dump files were downloaded from the NCBI FTP site. The BLAST outputs were parsed to retrieve the accession numbers of the hits, and then GenBank’s blastdbcmd program, along with the taxonomy dump files, were used to obtain the full taxonomic rank of each hit.

When a sequence read maps to multiple taxa, it is general practice to either ignore all hits for that read or divide the hit evenly across the taxa. The first option excludes taxa with homologous sequences, whereas the second option introduces false positives into the taxonomical assignments and alters abundance estimates. We chose the second option and mitigated false positives by excluding taxa with abundance counts of less than one.

During the analysis, a major update was made to the *Lactobacillus* taxonomical assignments, which included the parsing of *Lactobacillus* into 25 new genera (53). To accommodate this change, we updated the names of our final species-level taxonomical assignments using the NCBI Taxonomy Browser (https://www.ncbi.nlm.nih.gov/Taxonomy/TaxIdentifier/tax_identifier.cgi).

### Plasma metabolomics.

Approximately 0.5 to 1.0 mL of blood was drawn after the cessation of respiration via a closed-chest cardiac blood draw, using a tuberculin syringe (1.0 mL) and a 25-gauge needle. The aspirated blood was immediately transferred to a 2.0 mL EDTA tube at a rate of approximately 1 mL/30 s to mitigate hemolysis. The EDTA tube was inverted 10 times and was placed in wet ice. The EDTA blood tubes were processed within 1 h of placement into ice. Blood components were separated via centrifugation at 2,600 rpm (1,500 × *g*) for 10 min at 20°C. Plasma was carefully aspirated from the EDTA tube using a micropipette and aliquoted as 100 μL volumes into siliconized cryovials and stored at −80°C until metabolomic analysis. The small plasma volumes allowed for a single freeze-thaw cycle prior to the metabolomic analysis.

We performed targeted metabolomic measurements using stable isotope dilution multiple reaction monitoring mass spectrometry (SID-MRM-MS). We used the Biocrates AbsoluteIDQ P180 (BIOCRATES, Life Science AG, Innsbruck, Austria) Kit, as in our previous work (35–37). For this targeted analysis, frozen plasma samples were thawed on ice, vortexed, and processed as per the manufacturer’s instructions. The metabolite abundances were measured in a 96-well format, including seven calibration standards and three quality control samples that were integrated as part of the kit. The samples were analyzed with a triple quadrupole mass spectrometer (Xevo TQ-S, Waters Corporation, USA), operating in the multiple reaction monitoring (MRM) mode. The kit is capable of providing absolute quantitation for 21 amino acids, hexoses and carnitines, 39 acylcarnitines, 15 sphingomyelins, 90 phosphatidylcholines, and 19 biogenic amines. The flow injection analysis (FIA) tandem mass spectrometry (MS/MS) method was used to simultaneously quantify the 144 lipids via multiple reaction monitoring. The other metabolites were resolved via ultra-performance liquid chromatography (UPLC) and quantified using scheduled MRMs. The abundance of each metabolite was calculated from the area under the curve by normalizing to the respective isotope-labeled internal standard provided with the kit. The concentration of each metabolite is expressed in nmol/L. We used EDTA plasma samples spiked with standard metabolites as quality control samples to assess the reproducibility of the assay. The quantitated data were uploaded to RStudio, and values less than the limit of detection (LOD) were set to NA or 0, as appropriate for the specific analysis.

### Fecal metabolomics.

Fecal material was shipped on dry ice to the West Coast Metabolomics Center (WCMC) at UC Davis for extraction and analysis via hydrophilic interaction chromatography (HILIC) time-of-flight (TOF) mass spectrometry (MS). Standardized WCMC extraction and analysis protocols for “Biogenic amines by HILIC-QTOF MS/MS” were used throughout (analogous to those described by Ding [2021]) (54). Approximately 4 mg of fecal material were used from each sample. The final extract was dissolved in 100 μL acetonitrile/water (4:1, vol/vol) that contained internal control standards, and 3 μL of each extract was analyzed on a SCIEX 6600 TTOF, using both positive and negative ionization. We received the resulting data from the WCMC as a table containing ion identifications (where possible) and peak heights normalized to the sum of the internal standards.

### Microbiome analysis.

The processed sequence data were analyzed using RStudio Version 1.4.1106 (55) without rarefaction. Most of the graphics were created using the R package ggplot2 (56). The heat maps in Fig. 2 were generated from the standard normalized abundance data via the pheatmap package with “average” clustering (57). The Shannon diversity, nonmetric multidimensional scaling (NMDS), and permutational multivariate analysis of variance (PERMANOVA) were computed using the Vegan package (58). Both NMDS and PERMANOVA were applied to the Bray-Curtis dissimilarity matrix. PERMANOVA was performed using the Adonis function with 999 permutations and the following formula (SampleType and Cohort omitted for some analyses): data ~ Genotype + Sex + SampleType + Cohort + HousingID. Principal coordinates analysis (PCoA) was performed on the Bray-Curtis dissimilarities of the relative abundances using the cmdscale function from the stats package, and the resulting data were visualized with ggplot2 (56). Random forest was performed using the rfPermute package (v2.1.81) with a relative abundance threshold of 0.001. Linear mixed-effects modeling (LME) was performed on the relative abundance data using the lmer function in the lme4 package (46) with a relative abundance threshold of 0.001 and housing ID as the random effect.

### *Turicibacter* analysis with Bowtie2.

To determine the abundance of *Turicibacter* spp. in our samples, three reference genomes were downloaded from NCBI: *Turicibacter* sp. H121 (GenBank assembly accession: GCA_001543345.1), *Turicibacter* sp. HGF1 (GenBank assembly accession: GCA_000191865.2), and Turicibacter sanguinis (GenBank assembly accession: GCA_013046825.1). Bowtie2 v2.4.4 was used with the default parameters to align the reads from our samples to each *Turicibacter* reference genome (59, 60). The *Turicibacter* reads per sample were obtained and converted to relative abundances by using the total number of reads per sample. A Wilcoxon test was used for the significance testing in R v3.6.

### Metabolomics data analysis.

The analysis of both the fecal and plasma metabolomics data was performed using methods adapted from the microbiome analysis described above. The Spearman correlations between the metabolites and the species were generated using the cor function (method = spearman) from the stats package, and the resulting data were plotted using the pheatmap function from the pheatmap package (57).

### Data availability.

Raw sequence reads are available through the Sequence Read Archive (PRJNA902000). Both metabolomics and processed sequence data has been archived on Dryad (https://doi.org/10.7280/D1KH5N). The R scripts used to analyze all data are available on GitHub (https://github.com/sjbd1/5xfAD_mBio2022).
