# Supplementary material for: Longitudinal Analysis of the Microbiome and Metabolome in the 5xfAD Mouse Model of Alzheimer’s Disease
Source: mBio. 2022 Dec 5;13(6):e01794-22. doi: 10.1128/mbio.01794-22 (PMC9765021; doi:10.1128/mbio.01794-22)
Supplement: FIG S1 [file mbio.01794-22-s0002.pdf]

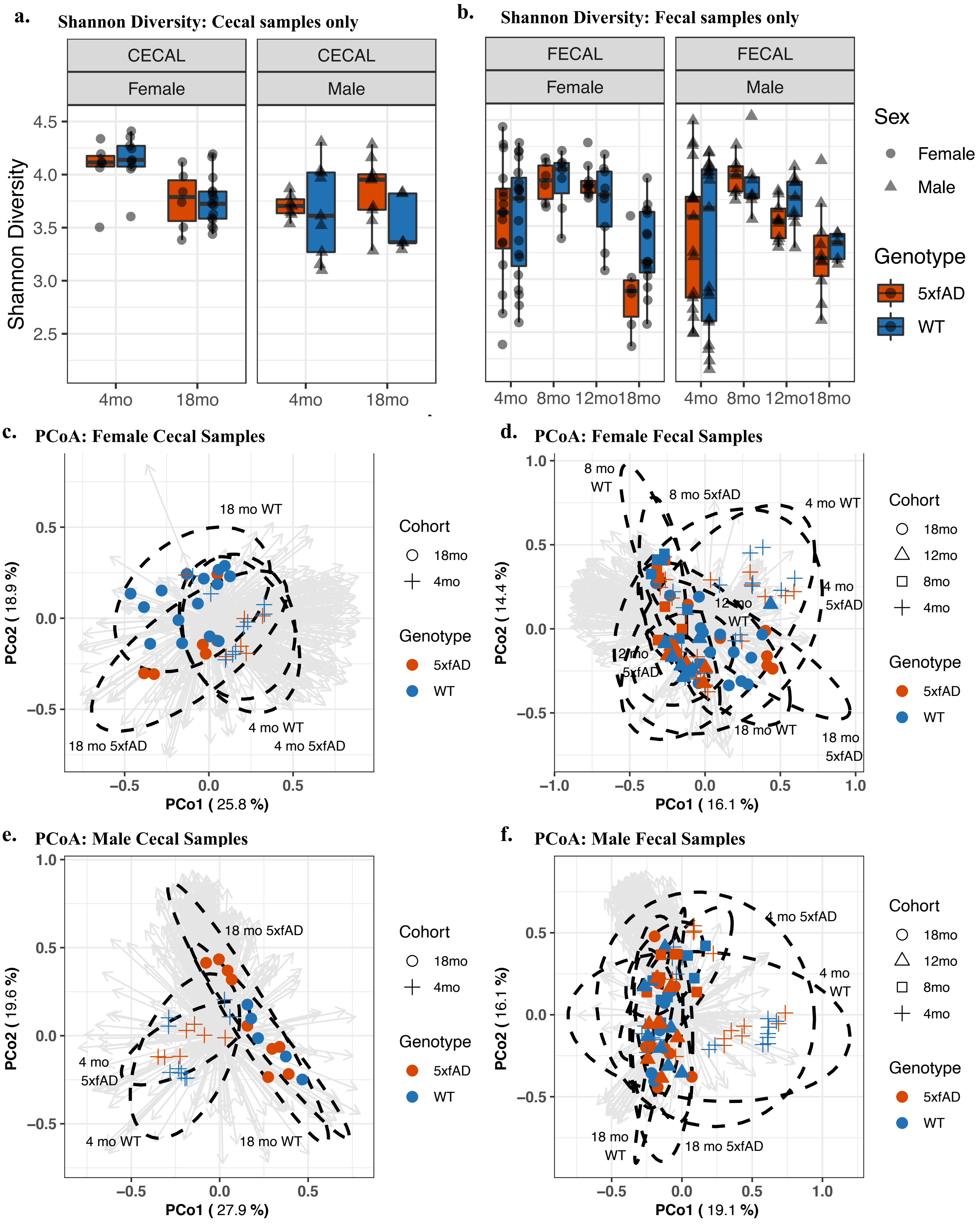

a.
Shannon Diversity: Cecal samples only
CECAL
Female
CECAL
Male
Shannon Diversity
4.5
4.0
3.5
3.0
2.5
4mo
18mo
4mo
18mo
b.
Shannon Diversity: Fecal samples only
FECAL
Female
FECAL
Male
4mo 8mo 12mo18mo
4mo 8mo 12mo18mo
Sex
Female
Male
Genotype
5xfAD
WT
c.
PCoA: Female Cecal Samples
18 mo WT
18 mo 5xfAD
4 mo WT
4 mo 5xfAD
PCo1 (25.8 %)
PCo2 (18.9 %)
Cohort
18mo
4mo
Genotype
5xfAD
WT
d.
PCoA: Female Fecal Samples
8 mo WT
8 mo 5xfAD
4 mo WT
4 mo 5xfAD
12 mo WT
12 mo 5xfAD
18 mo WT
18 mo 5xfAD
PCo1 (16.1 %)
PCo2 (14.4 %)
Cohort
18mo
12mo
8mo
4mo
Genotype
5xfAD
WT
e.
PCoA: Male Cecal Samples
18 mo 5xfAD
4 mo 5xfAD
4 mo WT
18 mo WT
PCo1 (27.9 %)
PCo2 (19.6 %)
Cohort
18mo
4mo
Genotype
5xfAD
WT
f.
PCoA: Male Fecal Samples
4 mo 5xfAD
4 mo WT
18 mo WT
18 mo 5xfAD
PCo1 (19.1 %)
PCo2 (16.1 %)
Cohort
18mo
12mo
8mo
4mo
Genotype
5xfAD
WT
