# Supplementary material for: Longitudinal Analysis of the Microbiome and Metabolome in the 5xfAD Mouse Model of Alzheimer’s Disease
Source: mBio. 2022 Dec 5;13(6):e01794-22. doi: 10.1128/mbio.01794-22 (PMC9765021; doi:10.1128/mbio.01794-22)
Supplement: TABLE S1 [file mbio.01794-22-s0001.pdf]

PERMANOVA Statistics Corresponding to Main Text Figure 3g							
**CECAL**							
	Df	SumsOfSqs	MeanSqs	F.Model	R2	Pr(>F)	
Genotype	1	0.1314	0.13142	6.027	2.3%	0.001	***
Sex	1	0.2619	0.26192	12.012	4.6%	0.001	***
Cohort	1	0.9925	0.99254	45.517	17%	0.001	***
Cage	21	3.4078	0.16228	7.442	60%	0.001	***
Residuals	41	0.894	0.02181		16%		
Total	65	5.6878			100%		
**FECAL**							
	Df	SumsOfSqs	MeanSqs	F.Model	R2	Pr(>F)	
Genotype	1	0.0782	0.07823	2.489	0.39%	0.027	*
Sex	1	0.5058	0.50583	16.09	2.5%	0.001	***
Cohort	3	3.3976	1.13253	36.025	17%	0.001	***
Cage	51	12.5791	0.24665	7.846	63%	0.001	***
Residuals	113	3.5524	0.03144		18%		
Total	169	20.1132			100%		
**Both Cecal & Fecal**							
	Df	SumsOfSqs	MeanSqs	F.Model	R2	Pr(>F)	
Genotype	1	0.1614	0.16139	5.667	0.60%	0.001	**
Sex	1	0.7444	0.74441	26.14	2.7%	0.001	***
Type	1	1.2861	1.28607	45.16	4.7%	0.001	***
Cohort	3	4.3819	1.46063	51.29	16%	0.001	***
Cage	51	15.4449	0.30284	10.634	57%	0.001	***
Residuals	178	5.069	0.02848		19%		
Total	235	27.0877			100%		

PERMANOVA Statistics Corresponding to Main Text Figure 3h							
**CECAL**							
**4 mo only**							
	Df	SumsOfSqs	MeanSqs	F.Model	R2	Pr(>F)	
Genotype	1	0.01749	0.01749	1.1621	0.89%	0.343	
Sex	1	0.42283	0.42283	28.0974	21%	0.001	***
Housing.ID	8	1.2319	0.15399	10.2326	62%	0.001	***
Residuals	20	0.30098	0.01505		15%		
Total	30	1.97319			100%		
**18 mo only**							
	Df	SumsOfSqs	MeanSqs	F.Model	R2	Pr(>F)	
Genotype	1	0.24614	0.24614	9.8614	9.0%	0.001	***
Sex	1	0.13137	0.13137	5.2631	4.8%	0.004	**
Housing.ID	12	1.84744	0.15395	6.168	68%	0.001	***
Residuals	20	0.4992	0.02496		18%		
Total	34	2.72415			100%		
**FECAL**							
**4 mo only**							
	Df	SumsOfSqs	MeanSqs	F.Model	R2	Pr(>F)	
Genotype	1	0.0276	0.02761	0.986	0.30%	0.393	
Sex	1	0.3926	0.39256	14.02	4.2%	0.001	***
Housing.ID	21	7.6169	0.36271	12.954	81%	0.001	***
Residuals	47	1.316	0.028		14%		
Total	70	9.353			100%		
**8 mo only**							
	Df	SumsOfSqs	MeanSqs	F.Model	R2	Pr(>F)	
Genotype	1	0.2061	0.206108	5.6352	2.9%	0.003	**
Sex	1	0.2705	0.270503	7.3958	3.8%	0.001	***
Housing.ID	23	5.2563	0.228533	6.2483	74%	0.001	***
Residuals	37	1.3533	0.036575		19%		
Total	62	7.0862			100%		
**12 mo only**							
	Df	SumsOfSqs	MeanSqs	F.Model	R2	Pr(>F)	
Genotype	1	0.07202	0.072017	2.8352	3.8%	0.007	**
Sex	1	0.12227	0.122271	4.8136	6.5%	0.001	***
Housing.ID	6	1.00207	0.167011	6.5749	53%	0.001	***
Residuals	27	0.68583	0.025401		36%		
Total	35	1.88219			100%		
**18 mo only**							
	Df	SumsOfSqs	MeanSqs	F.Model	R2	Pr(>F)	
Genotype	1	0.3393	0.33929	8.5487	8.3%	0.001	***
Sex	1	0.2236	0.22362	5.6341	5.5%	0.001	***
Housing.ID	12	2.6937	0.22447	5.6557	66%	0.001	***
Residuals	21	0.8335	0.03969		20%		
Total	35	4.0901			100%		
