# Supplementary material for: Longitudinal Analysis of the Microbiome and Metabolome in the 5xfAD Mouse Model of Alzheimer’s Disease
Source: mBio. 2022 Dec 5;13(6):e01794-22. doi: 10.1128/mbio.01794-22 (PMC9765021; doi:10.1128/mbio.01794-22)
Supplement: FIG S2 [file mbio.01794-22-s0003.pdf]

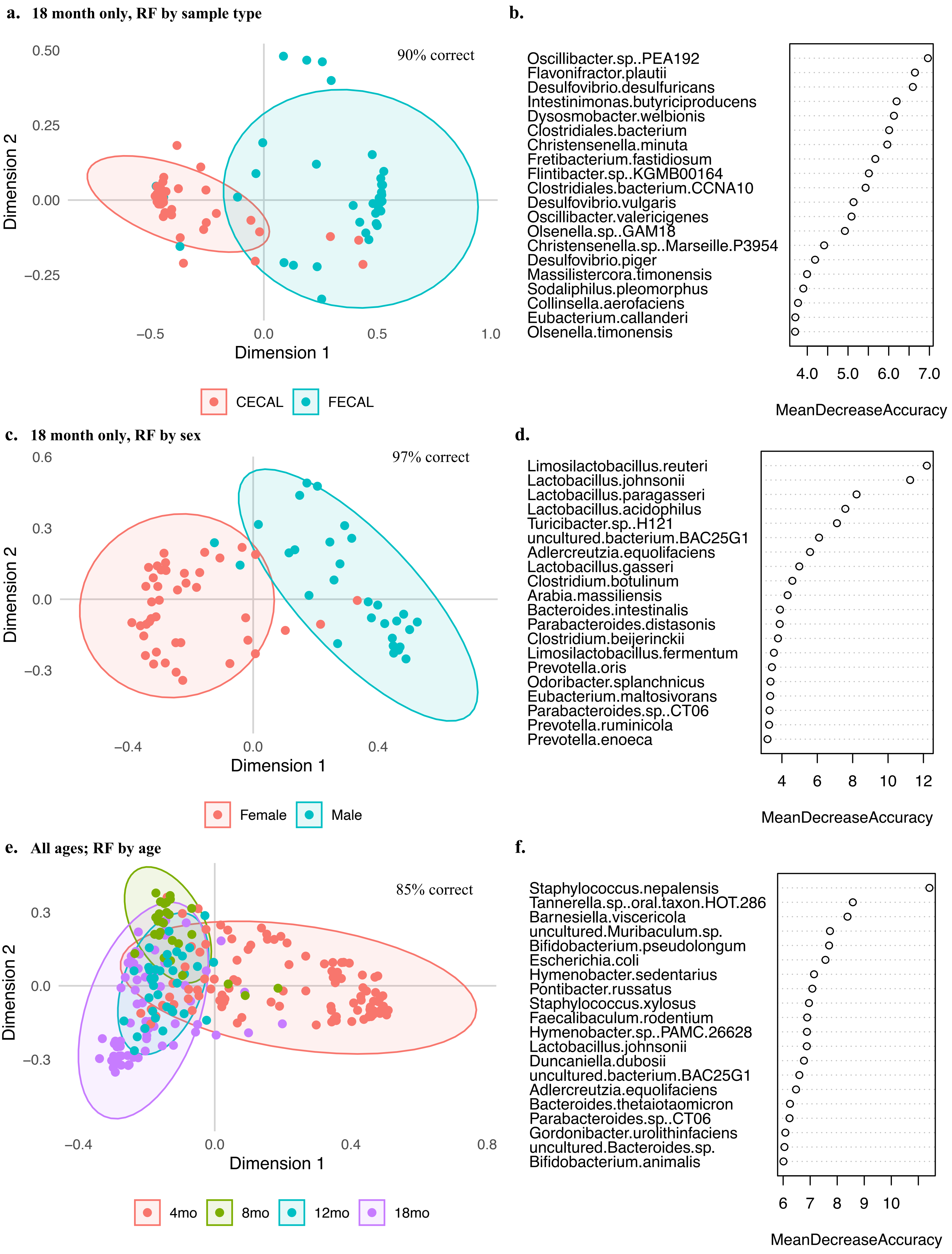
a. 18 month only, RF by sample type
90% correct
Dimension 2
Dimension 1
0.50
0.25
0.00
–0.25
–0.5
0.0
0.5
1.0
CECAL
FECAL
b.
Oscillibacter.sp..PEA192
Flavonifractor.plautii
Desulfovibrio.desulfuricans
Intestinimonas.butyriciproducens
Dysosmobacter.welbionis
Clostridiales.bacterium
Christensenella.minuta
Fretibacterium.fastidiosum
Flintibacter.sp..KGMB00164
Clostridiales.bacterium.CCNA10
Desulfovibrio.vulgaris
Oscillibacter.valericigenes
Olsenella.sp..GAM18
Christensenella.sp..Marseille.P3954
Desulfovibrio.piger
Massilistercora.timonensis
Sodaliphilus.pleomorphus
Collinsella.aerofaciens
Eubacterium.callanderi
Olsenella.timonensis
4.0
5.0
6.0
7.0
MeanDecreaseAccuracy
c. 18 month only, RF by sex
97% correct
Dimension 2
Dimension 1
0.6
0.3
0.0
–0.3
–0.4
0.0
0.4
Female
Male
d.
Limosilactobacillus.reuteri
Lactobacillus.johnsonii
Lactobacillus.paragasseri
Lactobacillus.acidophilus
Turicibacter.sp..H121
uncultured.bacterium.BAC25G1
Adlercreutzia.equolifaciens
Lactobacillus.gasseri
Clostridium.botulinum
Arabia.massiliensis
Bacteroides.intestinalis
Parabacteroides.distasonis
Clostridium.beijerinckii
Limosilactobacillus.fermentum
Prevotella.oris
Odoribacter.splanchnicus
Eubacterium.maltosivorans
Parabacteroides.sp..CT06
Prevotella.ruminicola
Prevotella.enoeca
4
6
8
10
12
MeanDecreaseAccuracy
e. All ages; RF by age
85% correct
Dimension 2
Dimension 1
0.3
0.0
–0.3
–0.4
0.0
0.4
0.8
4mo
8mo
12mo
18mo
f.
Staphylococcus.nepalensis
Tannerella.sp..oral.taxon.HOT.286
Barnesiella.viscericola
uncultured.Muribaculum.sp.
Bifidobacterium.pseudolongum
Escherichia.coli
Hymenobacter.sedentarius
Pontibacter.russatus
Staphylococcus.xylosus
Faecalibaculum.rodentium
Hymenobacter.sp..PAMC.26628
Lactobacillus.johnsonii
Duncaniella.dubosii
uncultured.bacterium.BAC25G1
Adlercreutzia.equolifaciens
Bacteroides.thetaiotaomicron
Parabacteroides.sp..CT06
Gordonibacter.urolithinfaciens
uncultured.Bacteroides.sp.
Bifidobacterium.animalis
6
7
8
9
10
MeanDecreaseAccuracy
