# Supplementary figures and images for: Longitudinal Analysis of the Microbiome and Metabolome in the 5xfAD Mouse Model of Alzheimer’s Disease
Source: mBio. 2022 Dec 5;13(6):e01794-22. doi: 10.1128/mbio.01794-22 (PMC9765021; doi:10.1128/mbio.01794-22)

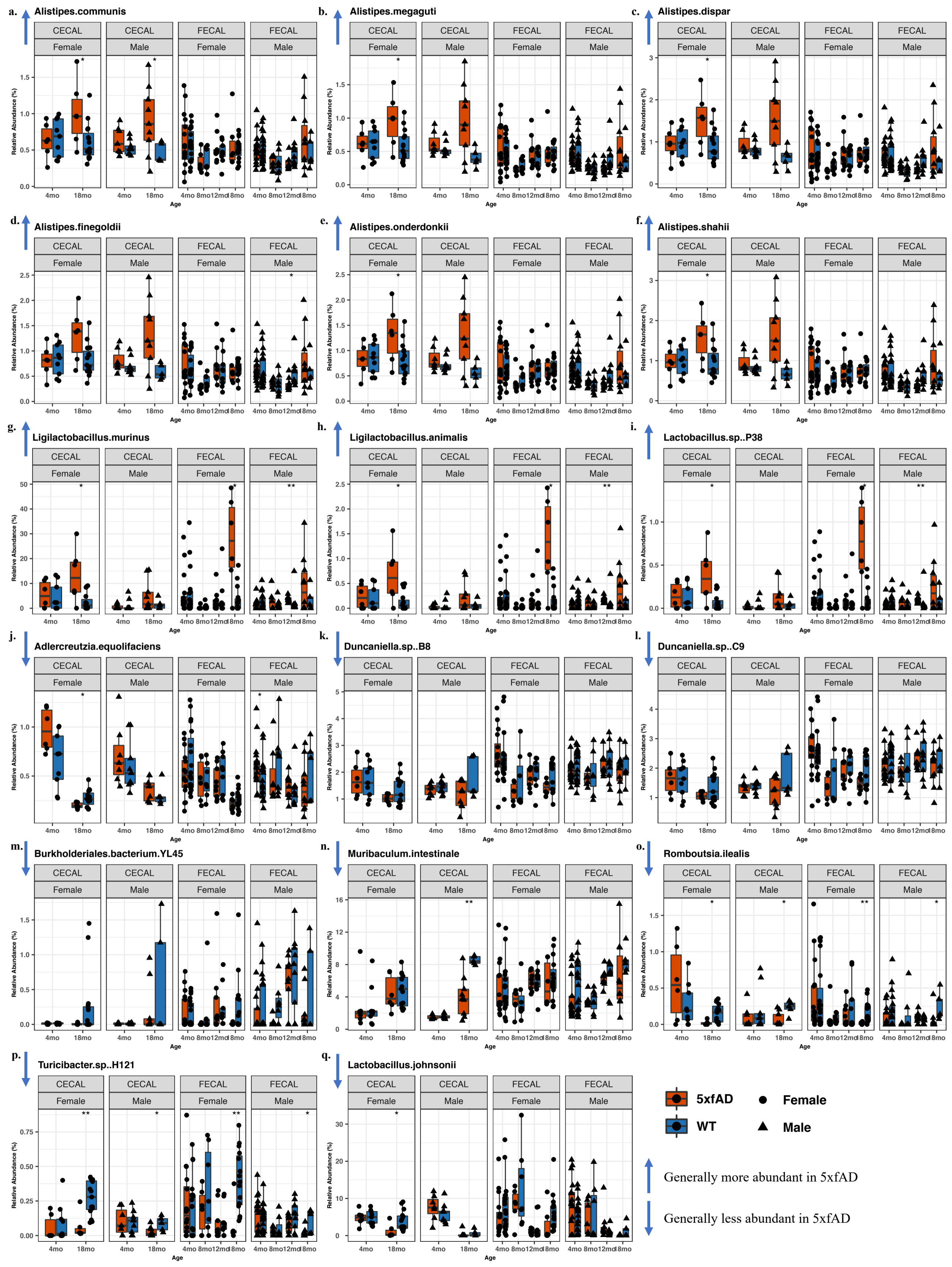

Supplement: FIG S3 [file mbio.01794-22-s0004.pdf]

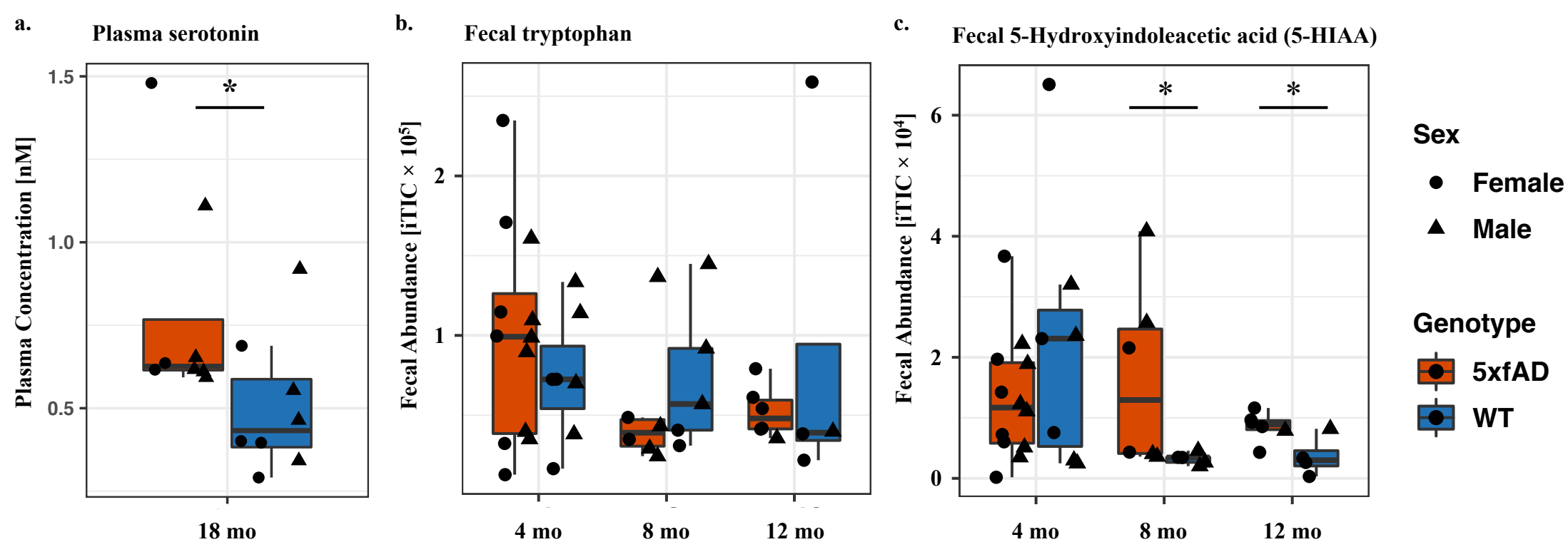

Supplement: FIG S7 [file mbio.01794-22-s0008.pdf]

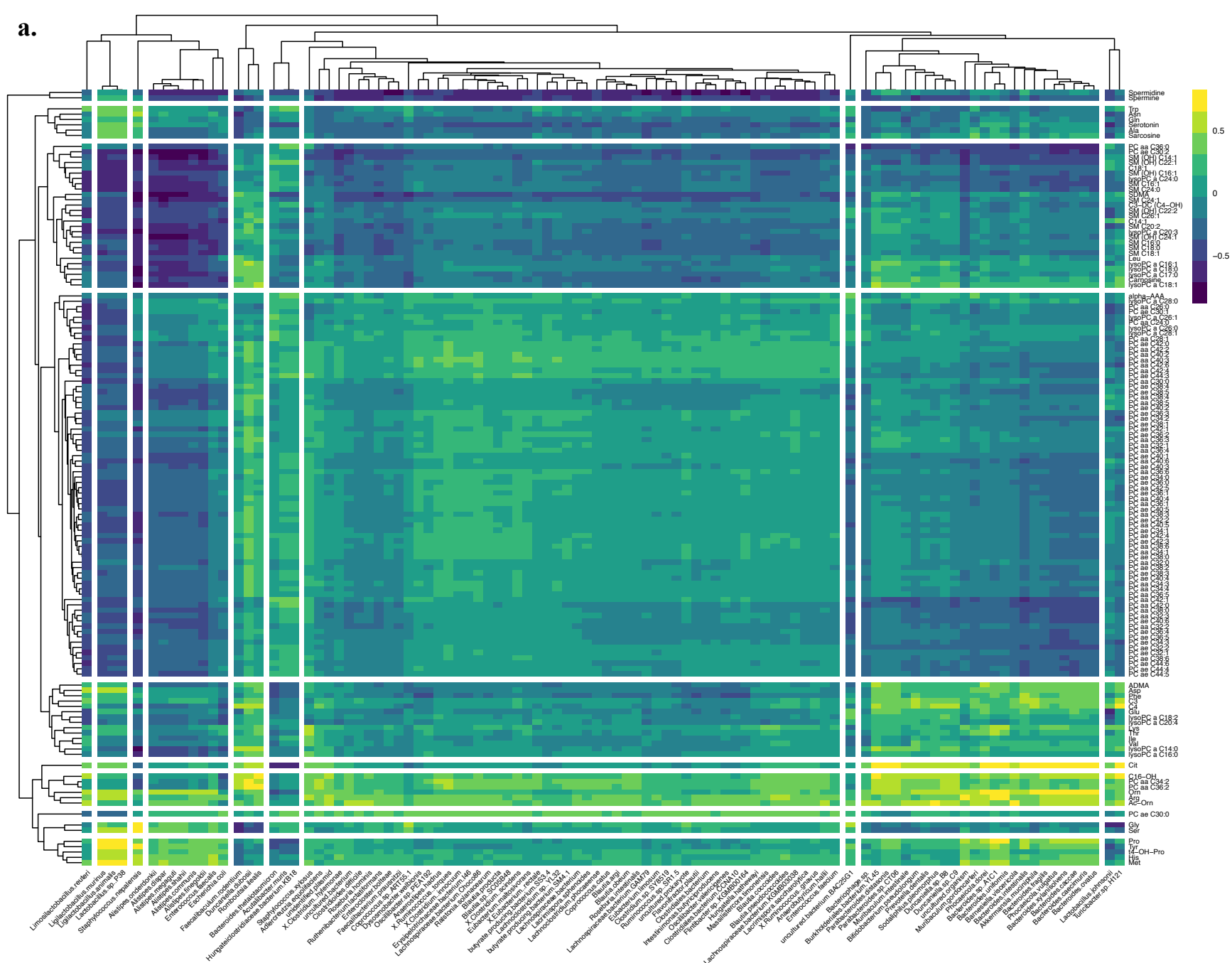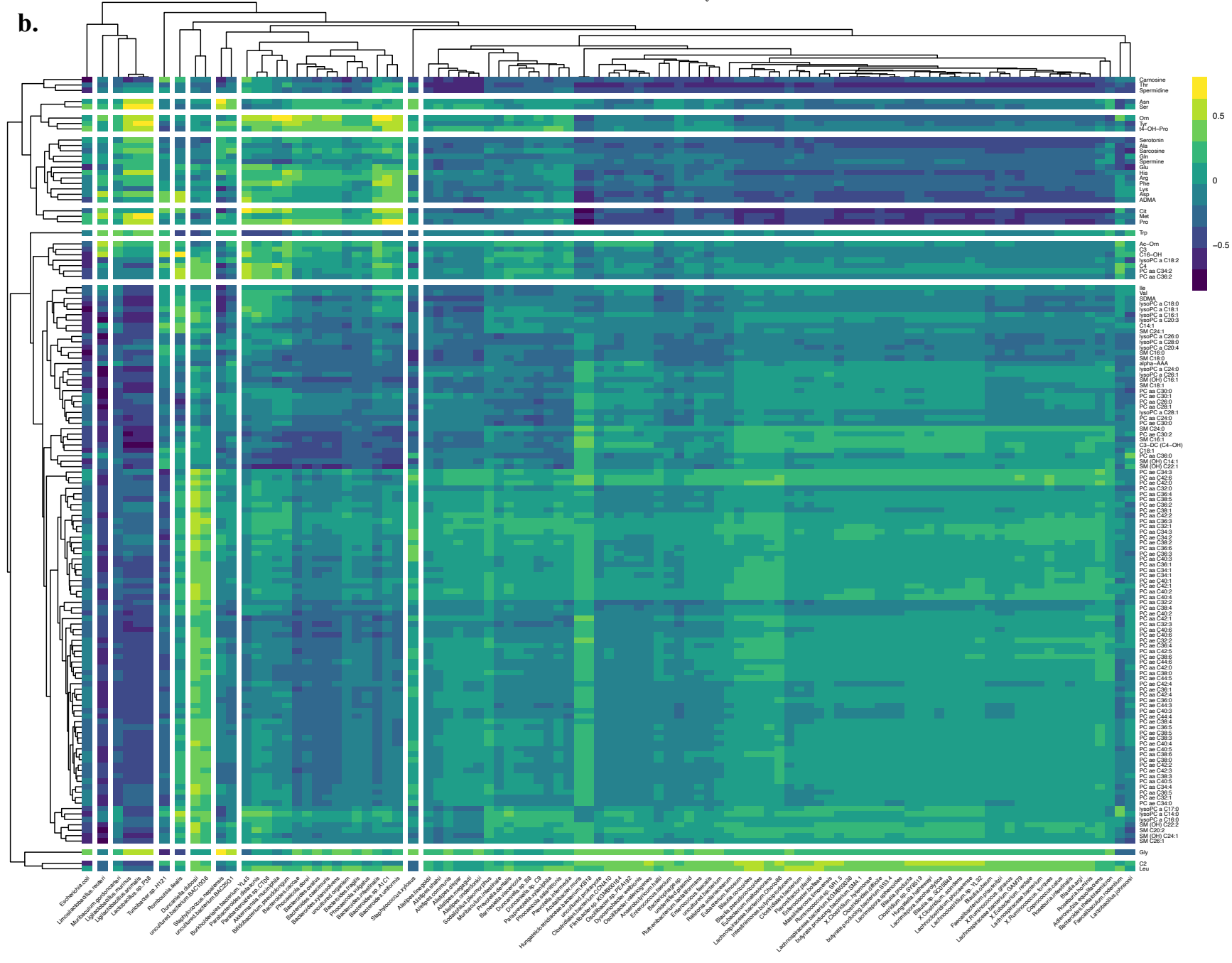

Supplement: FIG S8 [file mbio.01794-22-s0009.pdf]

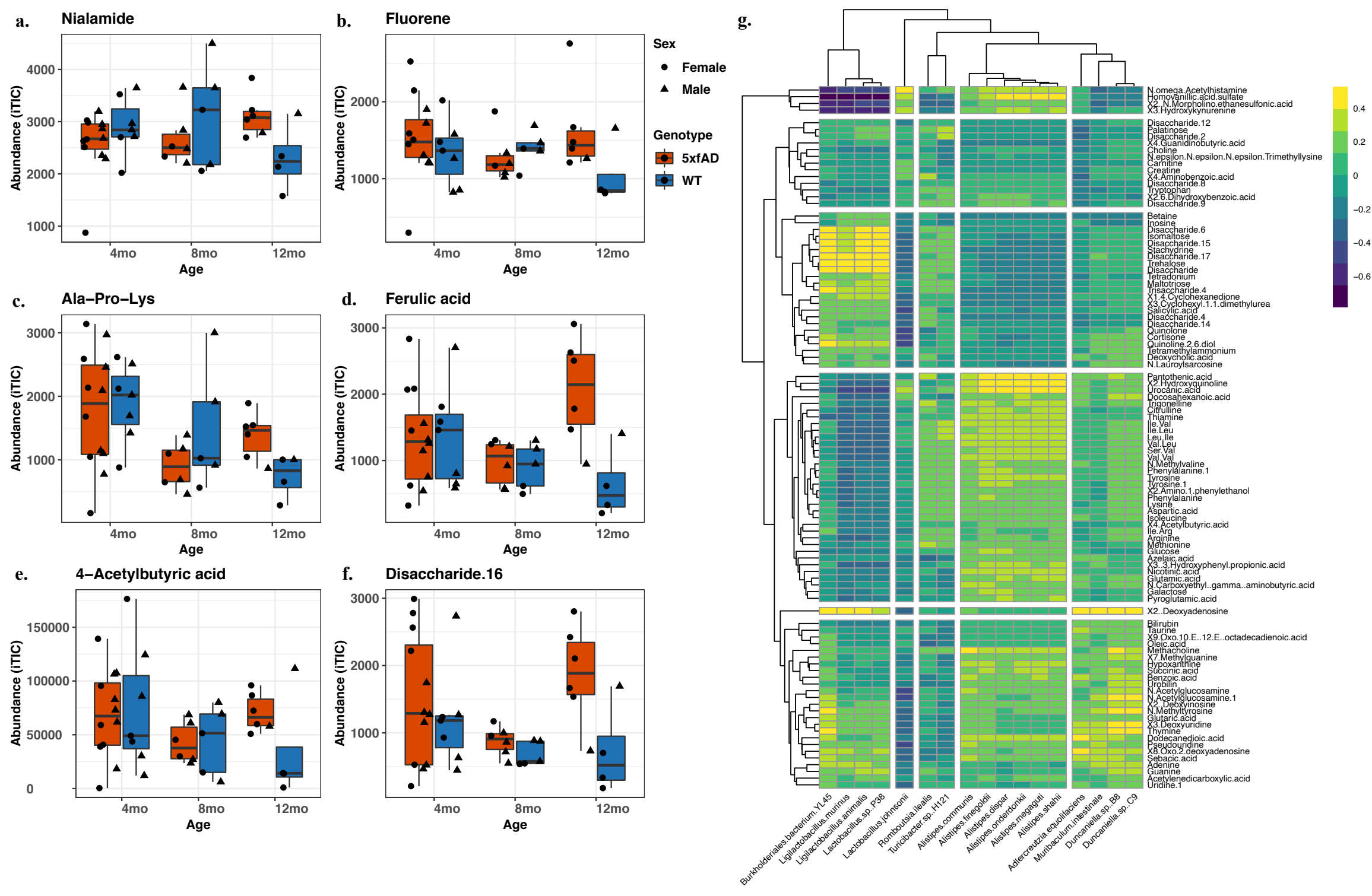

Supplement: FIG S9 [file mbio.01794-22-s0010.pdf]
