# Supplementary material for: Longitudinal Analysis of the Microbiome and Metabolome in the 5xfAD Mouse Model of Alzheimer’s Disease
Source: mBio. 2022 Dec 5;13(6):e01794-22. doi: 10.1128/mbio.01794-22 (PMC9765021; doi:10.1128/mbio.01794-22)
Supplement: FIG S4 [file mbio.01794-22-s0005.pdf]

a. Total Validated Metabolite Concentration

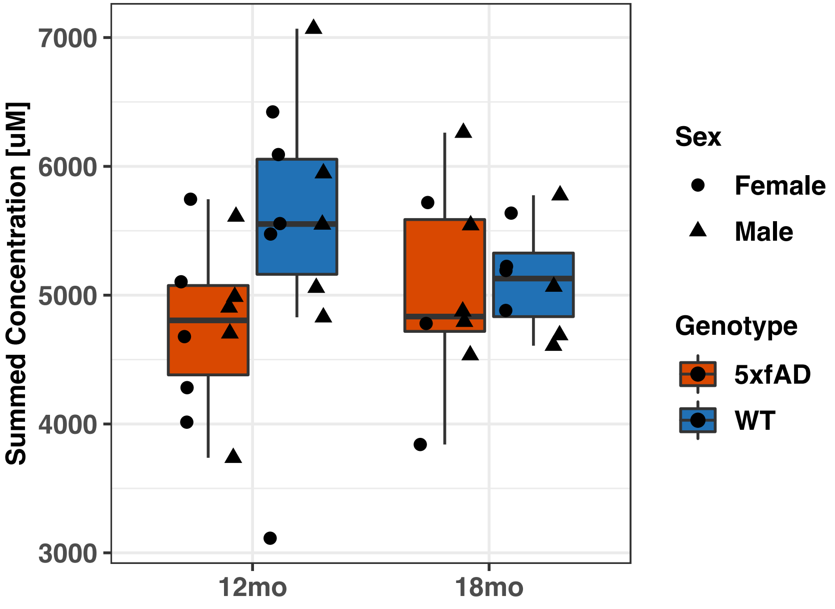

b. Shannon Diversity

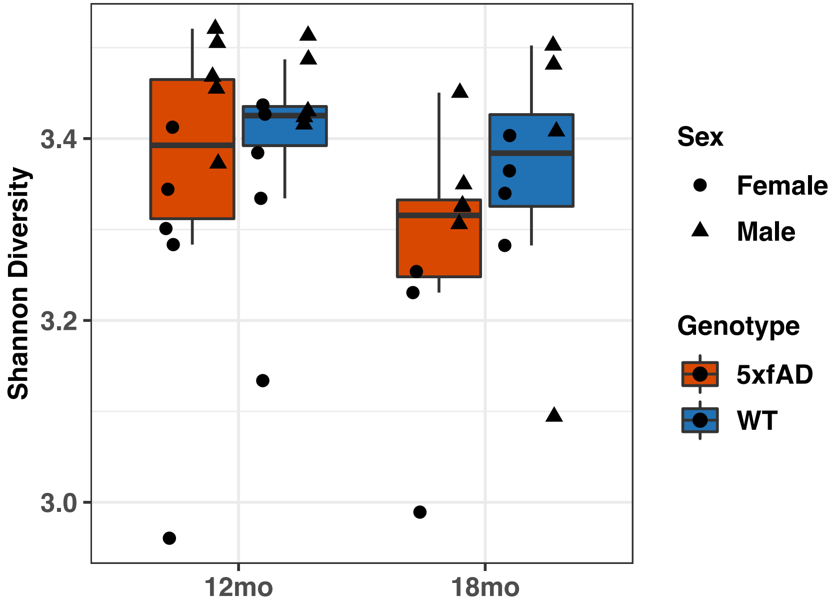

c. PCOA

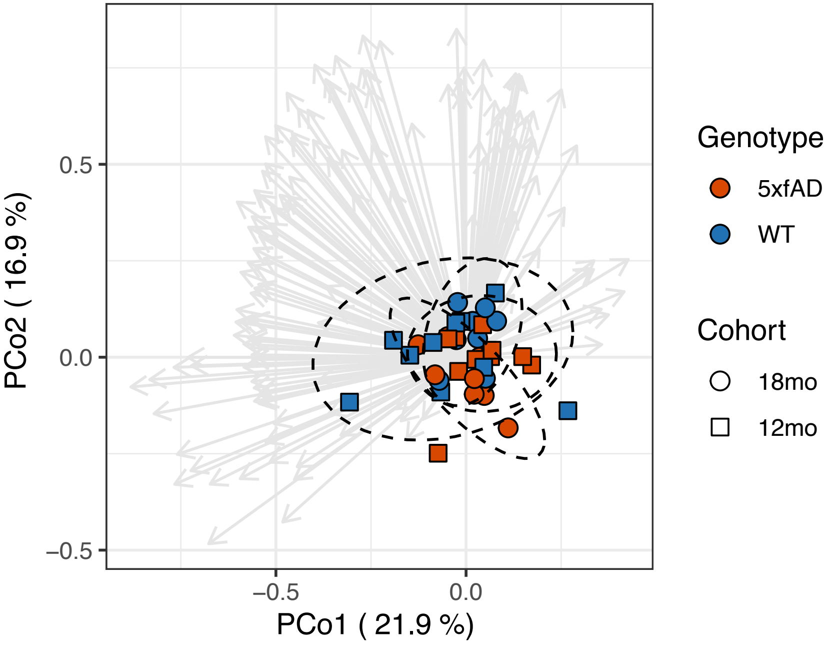

d. PERMANOVA

	Df	SumsOfSqs	MeanSqs	F.Model	R2	Pr(>F)
**Genotype**	1	0.2769	0.027690	1.90211	0.04891	0.104
**Sex**	1	0.03924	0.039241	2.69553	0.06931	0.037
**Age**	1	0.01423	0.014235	0.97782	0.02514	0.396
**Housing ID**	14	0.22293	0.015924	1.09382	0.39377	0.357
**Residuals**	18	0.26204	0.014558		0.46286	
**Total**	35	0.56614			1.00000	
