# Supplementary material for: Longitudinal Analysis of the Microbiome and Metabolome in the 5xfAD Mouse Model of Alzheimer’s Disease
Source: mBio. 2022 Dec 5;13(6):e01794-22. doi: 10.1128/mbio.01794-22 (PMC9765021; doi:10.1128/mbio.01794-22)

a. 12 and 18 month, by age

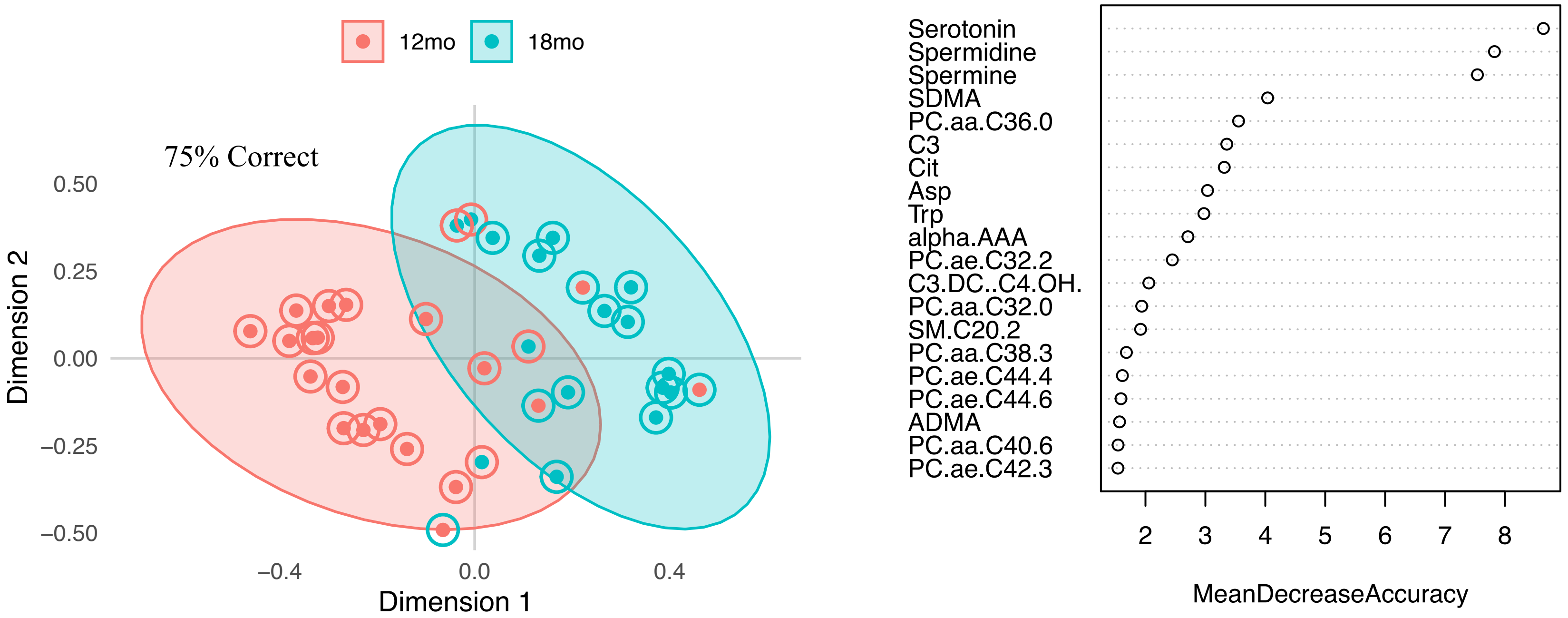

b. 12 and 18 month, by sex

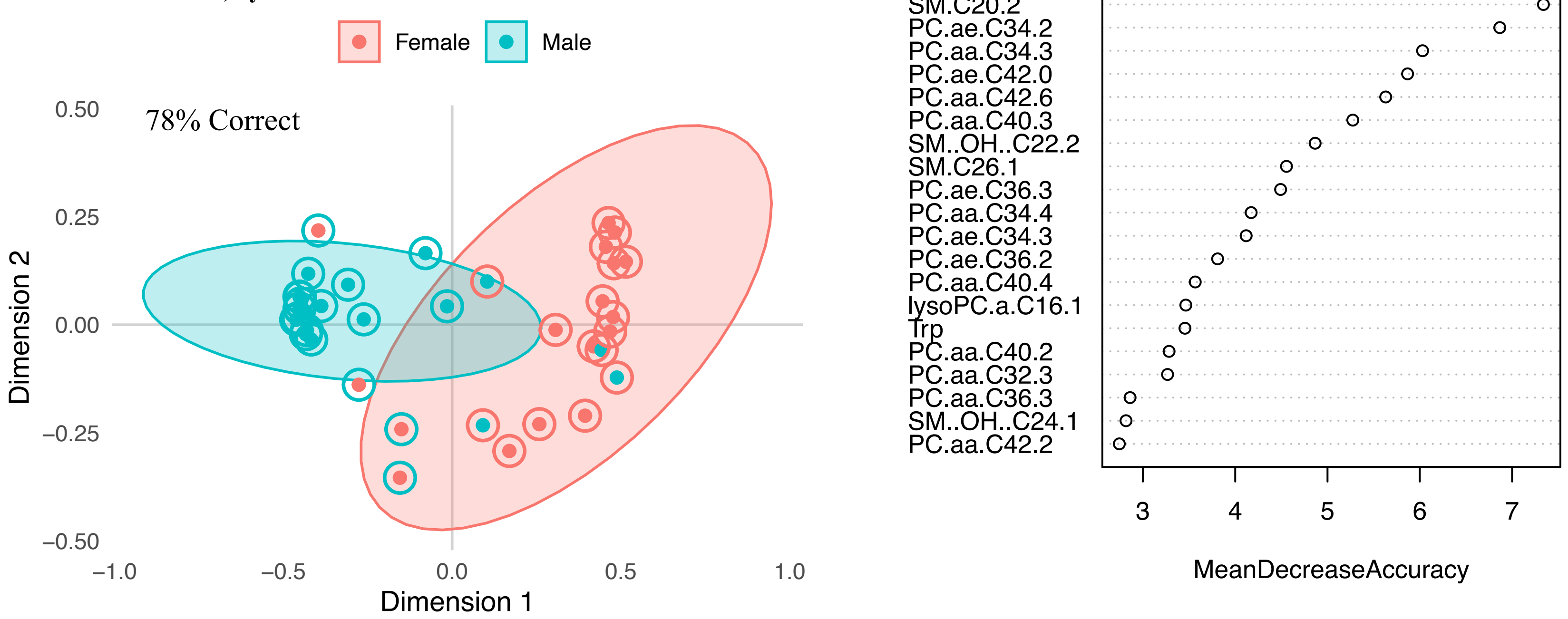

c. 18 month, by genotype

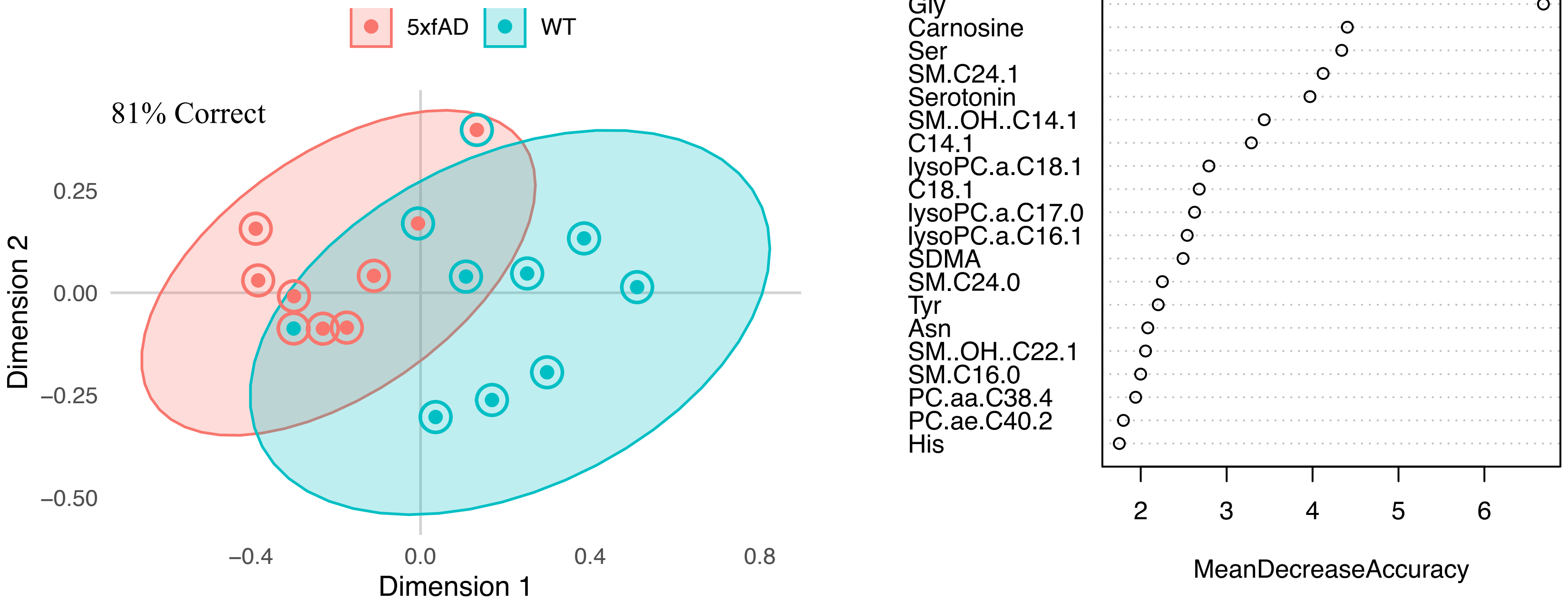

Supplement: FIG S5 [file mbio.01794-22-s0006.pdf]
