# Supplementary material for: Longitudinal Analysis of the Microbiome and Metabolome in the 5xfAD Mouse Model of Alzheimer’s Disease
Source: mBio. 2022 Dec 5;13(6):e01794-22. doi: 10.1128/mbio.01794-22 (PMC9765021; doi:10.1128/mbio.01794-22)

a.

18 month samples by genotype		
**Metabolite**	**padj**	**5xfAD vs WT**
Carnosine	<0.005	Lower in 5xfAD
**12 & 18 month samples by genotype**		
**Metabolite**	**padj**	**5xfAD vs WT**
Lyso PC a C18:1	<0.05	Lower in 5xfAD
Carnosine	<0.05	-
**12 & 18 month samples by age**		
**Metabolite**	**padj**	**18 vs 12 mo**
Serotonin	<0.005	Lower in 18 mo
Spermine	<0.05	Lower in 18 mo
Spermidine	<0.05	Lower in 18 mo
Glutamic acid	<0.05	Lower in 18 mo
**12 & 18 month samples by sex**		
**Metabolite**	**padj**	**F vs M**
SM C20:2	<0.005	Lower in F
PC ae C42:0	<0.01	Lower in F
PC aa C34:3	<0.05	Lower in F
PC aa C42:6	<0.05	Lower in F
Lysine	<0.05	Lower in F
SM (OH) C24:1	<0.05	Higher in F
PC aa C36:4	<0.05	Lower in F
PC aa C36:5	<0.05	Lower in F
PC ae C38:0	<0.05	Lower in F
PC aa 36:3	<0.05	Lower in F
PC aa 38:6	<0.05	Lower in F

b.

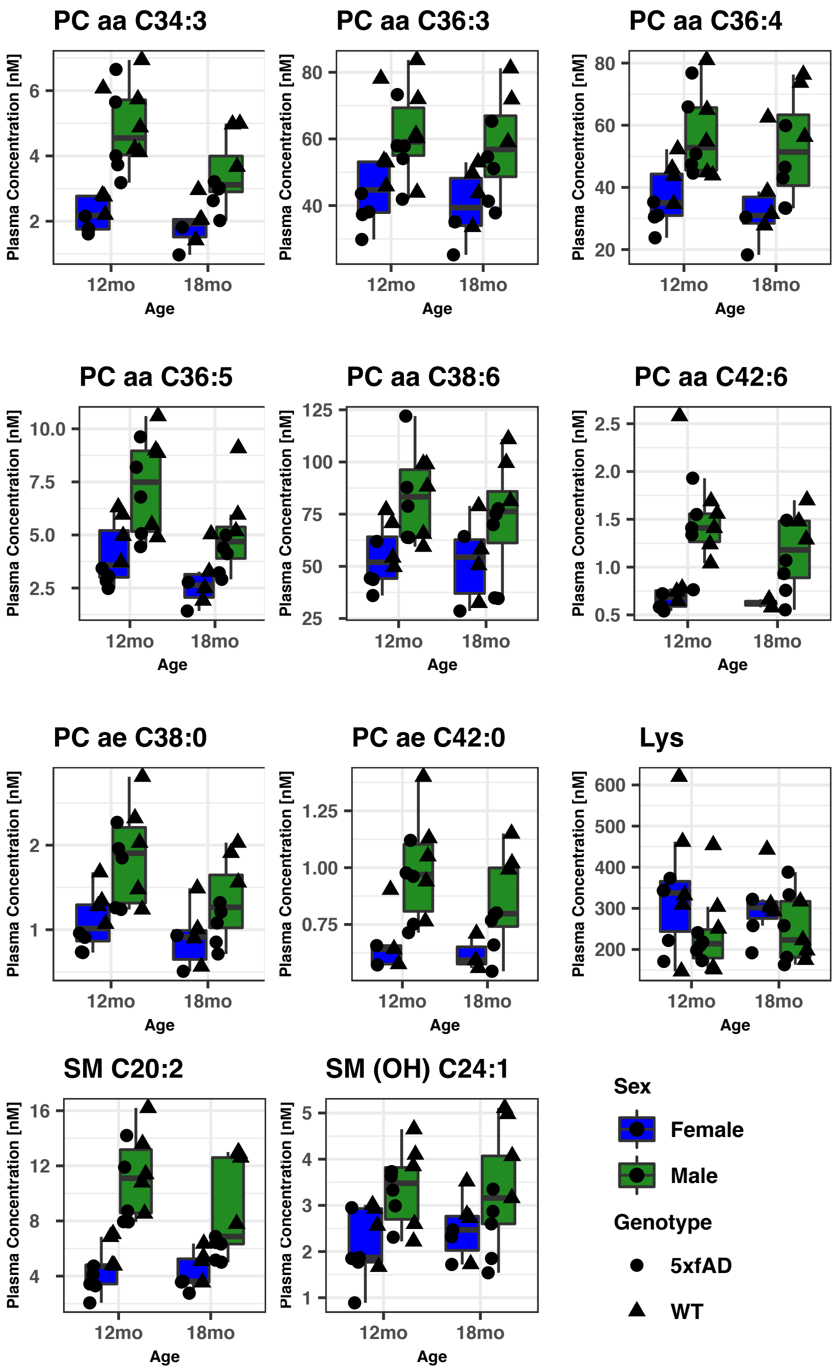

Supplement: FIG S6 [file mbio.01794-22-s0007.pdf]
